# PLAE Web App Enables Powerful Searching and Multiple Visualizations Across One Million Unified Single-Cell Ocular Transcriptomes

**DOI:** 10.1167/tvst.12.9.18

**Published:** 2023-09-25

**Authors:** Vinay S. Swamy, Zachary A. Batz, David M. McGaughey

**Affiliations:** 1 Department of Biomedical Informatics, Columbia University, New York, NY, USA; 2 Bioinformatics Group, Ophthalmic Genetics and Visual Function Branch, National Eye Institute, National Institutes of Health, Bethesda, MD, USA; 3 Neurobiology, Neurodegeneration and Repair Laboratory, National Eye Institute, National Institutes of Health, Bethesda, MD, USA

**Keywords:** single cell, app, resource

## Abstract

**Purpose:**

To create a high-performance reactive web application to query single-cell gene expression data across cell type, species, study, and other factors.

**Methods:**

We updated the content and structure of the underlying data (single cell Eye in a Disk [scEiaD]) and wrote the web application PLAE (https://plae.nei.nih.gov) to visualize and explore the data.

**Results:**

The new portal provides quick visualization of over a million individual cells from vertebrate eye and body transcriptomes encompassing four species, 60 cell types, six ocular tissues, and 23 body tissues across 35 publications. To demonstrate the value of this unified pan-eye dataset, we replicated known neurogenic and cone macula markers in addition to proposing six new cone human region markers.

**Conclusions:**

The PLAE web application offers the eye community a powerful and quick means to test hypotheses related to gene expression across a highly diverse, community-derived database.

**Translational Relevance:**

The PLAE resource enables any researcher or clinician to study and research gene expression patterning across a wide variety of curated ocular cell types with a responsive web app.

## Introduction

The outer layer of the eye consists of the cornea and sclera. Beneath the sclera is the uvea, which is comprised of the iris, ciliary body, and choroid. The inner most layer of the eye is the retina, which is backed by the retinal pigmented epithelium (RPE). Distinct cell types are used to compose each of these layers. The retina alone is composed of six major cell populations: the photoreceptors, horizontal, bipolar, amacrine, ganglion, and non-neuronal cells.^1^ These six populations can be subdivided into dozens of cell types.^2^ Furthermore, human disease of the eye can derive from damage to specific cell types.

Damage to some of these cell types has been implicated in human eye disease. For example glaucoma is characterized by damage to the retinal ganglion cells, and cone and cone–rod dystrophies are characterized by photoreceptor degeneration. Efforts to untangle the gene expression patterns of these diverse cell types with single-cell transcriptomics date to the early 2000s when researchers^3^^–^^7^ used reverse transcription polymerase chain reaction (RT-PCR) approaches to amplify select transcripts in small numbers of cells. Later, researchers used microarray platforms to analyze a broader array of transcripts.^8^^–^^14^

The modern era of single-cell transcriptomics started with the introduction of droplet-based technologies, which enabled quantitation of thousands of cells in a single experiment.^15^ This approach was pioneered by Macoksko et al.^16^ and enabled the profiling of gene expression in 44,808 single cells in the adult mouse retina. Commercialization of the droplet technology has enabled broad access, and the ocular community has responded by using single-cell RNA sequencing in several dozen ocular studies. An incomplete list of how the ocular community has used single-cell technology would include studying developmental transcriptomic dynamics, identifying genes to distinguish cell types, proposing finer gradations of cell types within existing categories, comparing and contrasting ocular transcriptomes between organisms, and identifying differences in spatially distinct cells.

Unfortunately, manuscripts can only present a sliver of the underlying data. Further data exploration can be laborious, technically challenging, and computationally expensive. Briefly, one would have to identify the relevant publication, find the associated data deposit, download the cell by gene count matrix, find the cell label table (and optionally t-distributed stochastic neighbor embedding [t-SNE] or Uniform Manifold Approximation and Projection [UMAP] coordinates) with the matching fields to align them, and then load the data into R or Python to run the desired queries. If one desired to compare across publications, then one would also have to align the cell-type label schema and ideally should recreate the count quantification with consistent bioinformatic tooling. It is desirable, perhaps even necessary, to use multiple datasets to assess gene expression to confirm whether a gene expression pattern is consistent and reproducible. Reliance on a single dataset can be problematic, as there can be technical issues; for example, the large consortium dataset GTEx has persistent contamination from highly expressed genes across tissues.^17^

Web-based resources to query gene expression can democratize access to large datasets as the computationally intensive steps above can be run once and then shared to anyone with a internet connected device. Single-cell RNA sequencing (RNA-seq) web resources that contain ocular tissues include the University of California, Santa Cruz (UCSC) Cell Browser, Tabula Sapiens, PanglaoDB, and the Protein Atlas.^18^^–^^21^ However, ocular-focused queries are challenging, as these resources have incomplete and inconsistent cell-type labels and sample metadata. In contrast to there general purpose resources, Spectacle (http://singlecell-eye.org) is an ocular specific web app that contains over a million cells from dozens of independent resources.^22^ However, each data deposit is independently and inconsistently processed, which makes cross-study queries impossible.

We recently created a unified ocular data resource that placed dozens of independent single-cell RNA-seq ocular and non-ocular datasets in a single-batch corrected space, the single cell Eye in a Disk (scEiaD).^23^ To broaden the usage of this powerful meta-atlas dataset, we restructured the data into a custom sqlite-based database and built a reactive web application to access it. As many new studies were published since our last data build, we re-ran the scEiaD pipeline in March of 2022. In aggregate, we have collated 44 datasets across 35 publications, four species, and 29 tissues. We hand-curated 60 published cell type labels, totaling 606,612 cells, and used those ground-truth labels as the basis for a machine learning–based algorithm that labels the 500,018 previously unannotated cells. Quality control metrics were tuned and applied to remove lower quality cells. In the end, the scEiaD database contains 1,136,041 cells and 1,509,179,347 non-zero gene/cell expression values. We demonstrate how this unified resource can be used to identify genes that consistently distinguish cell types across studies and species, find genes that have species-specific expression cell type patterning, demonstrate how our pre-computed differential testing can identify markers between similar progenitor cell types, and finally provide an analysis document based on R (R Foundation for Statistical Computing, Vienna, Austria) that end-to-end demonstrates how we have used scEiaD to run a custom differential gene expression test to validate and propose new genes that distinguish macula from peripheral cones in humans.

## Methods

### scEiaD Pipeline Upgraded and the Database Rebuilt

Our full scEiaD pipeline was published previously in Swamy et al.^23^ It was composed of two Snakefiles that run the workflows.^24^ The SnakeQUANT workflow takes the raw fastq input and produces a gene by cell count matrix with kallisto and bustools.^25^^,^^26^ This matrix is used in the SnakePOP pipeline to identify the optimal parameters for the scANVI-based batch correction process (Supplementary Fig. S1). Outputs from this pipeline include the batch-corrected lower dimensional space, the UMAP coordinates, cell cluster assignments, and the cell-type predictions. The outputs from this pipeline are used in a our new Snakefile SnakeSCEIAD which performs differential gene testing across multiple conditions and wraps the various data types into the scEiaD sqlite database for use in the plaeapp (Supplementary Fig. S2).

### Quality Control

To decide whether a droplet could be considered a viable cell, we used the barcodeRanks algorithm from DropletUtils to automatically identify empty droplets.^27^ We then kept cells with the following criteria: less than 10% of the counts were in mitochondrial genes (20% for 10X v3, as this platform has overall higher mitochondrial counts), and a cell had more than 600 total counts, at least 300 unique genes quantified, and fewer than 100,000 total counts. To identify likely doublets, we used Scrublet^28^ and doubletCell (from the R package scran)^29^ to generate per-cell doublet probability scores. To identify cells for removal we used the scores in two ways. First, if an individual cell had a Scrublet score greater than 0.8 (where 1.0 is 100% probability of doublet) and a doubletCell score greater than 1,000,000 (unitless measure where higher value is more likely to be doublet), the cell was labeled as a doublet. Second, if a cluster had an average Scrublet score greater than 0.2 or an average doubletCell score greater than 12, then the entire cluster was marked as doublets. All cutoff values were selected by hand-inspecting density distributions of the Scrublet and doubletCell scores. Study-level distributions of percent mitochondrial genes, total gene counts, and unique genes identified are shown in Supplementary Figure S3.

### Alterations From Swamy et al. to Increase Stringency of the Dataset

The following alterations were made to the Swamy et al.^23^ pipeline: First, the cutoff to retain a cell was raised from 200 quantified unique genes to 300. Second, we use the DecontX algorithm^30^ from the celda R package (v1.9.2) to automatically remove ambient RNA contamination on a per-study basis to produce a updated count matrix. Third, we altered the procedure for aligning gene names across species (see below for details). Fourth, we used the scANVI (scvi-tools based) batch correction method, which leverages information from the known cell-type labels in the batch correction process.^31^ A custom cutoff for the minimum required number of unique genes identified per cell was used for the SRP362101 cornea dataset, as an abnormally high number of cells were returned from our default value of 300. We set the cutoff for this study to 800, as this value resulted in approximately the same number of cells being returned as the authors reported in their paper.

### Optimal Parameter Choices for scANVI Batch Correction

We found in Swamy et al.^23^ that scVI had the best batch integration performance relative to other tools, such as Seurat's canonical correlation analysis (CCA), fastMNN, harmony, ComBat, scanorama, and others. We previously found that adjusting the number of latent dimensions, highly variable genes, and epochs had substantial effects on the integration performance. For this update of scEiaD, we ran integration with 3000, 4000, 5000, and 6000 highly variable genes; five and 15 epochs; and six, eight, 10, 15, and 20 scANVI outputted latent dimensions. After evaluating the performance of the new scEiaD dataset with our scPOP package, we selected the following parameters for the integration: 15 latent dimensions, 4000 highly variable genes, and five epochs.^23^ The UMAP was built with a min_dist of 0.1, and the clustering used 50 nearest neighbors.

Although nearly all datasets we curated could be integrated as a whole, a small number either did not integrate with other data (clusters were formed which were entirely composed of one study) or nearly all cells from a study were machine labeled as a single cell type. These studies were hand removed from the resource. The metadata for these samples are available as Supplementary Material (samples_hand_removed.csv).

### Cross Species Gene Alignment

As we had four species in scEiaD (*Gallus gallus*, *Macaca fascicularis*, *Mus musculus*, and *Homo sapiens*) that we unified, we had to identify shared genes. We found homologs and orthologs by downloading from the BioMart database Ensembl Genes 105 and using *Homo sapiens* as the reference. We then selected orthologous gene names from *Gallus gallus*, *Macaca fascicularis*, and *Mus musculus*. This generated a table linked by Ensembl gene IDs and gene names. We then used a full join on non-duplicated Ensembl IDs between mouse and human. As we noticed a small number of gene names failed to be linked in this manner, we ran another join on the remaining non-aligned genes, using gene name. This procedure gave us 17,769 shared human and mouse genes. To align the other species we again joined on Ensembl gene ID, removing genes where one chicken or macaque gene aligned to multiple human/mouse genes. In cases where multiple chicken or macaque genes aligned to one human/mouse gene, then we aggregated the counts by sum. In cases where there was no corresponding chicken or macaque gene, we filled in zeros. After these steps, we had, as expected, 17,769 genes.

### Metadata Curation

Every study brought into scEiaD was hand curated to identify the unique biological samples, published paper ID (where available), organ, tissue (e.g., cornea), source (tissue), single-cell platform (e.g., 10X v2, DropSeq). We also, where possible, annotated retina region (e.g., macula), sex, and age or developmental stage. Samples with perturbations (genetic or treatment) were not included. To import individual cell-type labels (e.g., rod, Müller glia), we wrote custom code for each study that made this table available to link their cell-type assignments back to the matching barcoded cell. We curated two types of cell type labels: CellType and SubCellType, where the former is our normalized cell types taken from the published labels and the latter are the original cell-type labels. We normalized names for CellType—for example, changing “MG” to “Muller Glia” and dropping more detailed cell type assignments, such as off and on bipolar cells for Bipolar Cells (as few studies went into this level).

### Differential Gene Testing

We used a pseudo-bulk–based approach where the counts for a category (CellType, Cluster, or CellType [Predict]) were summed for each category–organism–study grouping using the aggregateAcrossCells function from the scuttle R package.^32^ This created a counts matrix that had statistical properties approximating bulk RNA-seq experiment matrices. This allowed use of the more mature bulk RNA-seq tooling. This approach has also been found to be more specific in identifying differentially expressed genes in single cell RNA-seq experiments.^33^ If there were fewer than 50 cells in organism–study accession–category combination, they were discarded for the differential test. Any genes with a sum of 0 counts after aggregation were removed. The aggregated matrix was imported into DESeq2 using the DESeqDataSetFromMatrix function with the design given as “∼study_accession + category.” Contrasts were extracted either with the target category (e.g., Cone) against all remaining or in a pairwise manner (Cone vs. Rod) with the DESeq2 results function.^34^ We tested each species (human, macaque, and mouse) separately.

### PubMed Citation Search

The R package easyPubMed was used to search for 1000 randomly chosen genes present in scEiaD for both gene name alone and gene name plus “AND Retina” with the get_pubmed_ids function. We then used the returned Entrez identifier to pull the abstract with the fetch_pubmed_data function so we could extract the PubMed ID (PMID). This was repeated with the genes identified as being well supported differentially expressed for ocular cell types. To assess whether the difference in distributions was statistically different, we used the base R t.test function.

### scEiaD Structure and Web App Optimization

The web app at plae.nei.nih.gov can display gene expression across over a million cells in only a few seconds. This speed is only possible due to custom data structures that were optimized for data query efficiency. Like the eyeIntegration EiaD database,^35^ scEiaD is a sqlite database with tables for the core categories. This allows the app to initialize in about 30 seconds on a cloud server with memory usage under 8 GB. For the gene-by-cell expression matrix, the data were transformed into a “long” format with three columns: gene, cell barcode, and gene count value. This allowed for cell–gene level queries to be completed, again with minimal memory usage, in under 0.5 seconds. To generate the aggregated information (in the Dot Plot, Expression Plot, and tables) we pre-calculated all queries by running a dplyr “group by” operation on all column fields. Upon a user-given query, the data are further aggregated to the user’s request. This allows for complicated queries to complete in seconds rather than minutes. To compare web app loading times for useful tasks we queried https://plae.nei.nih.gov, http://singlecell-eye.org, and http://tabula-sapiens-portal.ds.czbiohub.org on August 24, 2022. For Spectacle, we used the “Single-Cell RNA-Seq Analysis of Retinal Development” 10X dataset from Clark et al.^49^ for all timings. Timings are accurate to the second. If the functionality tested was not available for a certain web app, it was represented by a blank space.

### Data Reproducibility and Availability

The code base for scEiaD is available at github.com/davemcg/scEiaD; the commit corresponding to this manuscript is #a99dced. There are three relevant Snakemake pipelines that were used to create scEiaD: SnakeQUANT, which was used to quantify the gene/cell expression from the raw fastq files; SnakePOP, which runs the multi-parameter integration methods; and SnakeSCEIAD, which uses the optimal parameters identified in SnakePOP to run the cell-type machine learning and differential gene testing that are incorporated into the scEiaD sqlite database for the app. The code base for the web app (along with local installation instructions) is available at https://github.com/davemcg/plaeApp. Data are available for download at plae.nei.nih.gov (click “Data”), and the Seurat object and metadata for the resource have been deposited at Zenodo with accession 10.5281/zenodo.7071682. Scripts associated with this paper's analysis can be found at https://github.com/davemcg/plaeApp_manuscript; the associated commit is #2063e53 and Zenodo with accession 10.5281/zenodo.8349725.

## Results

### New Studies and Processing Improvements Enhance the scEiaD Database

Our first version of the single cell Eye in a Disk (scEiaD) database contained 34 datasets, three species, 766,615 cells, and 31 curated cell types.^23^ We updated the scEiaD database for the PLAE v0.94 web app in six important ways. First, we added a new species, chicken (*Gallus gallus*).^36^ Second, we added two ocular outflow tract datasets, one brain choroid plexus, and three cornea datasets to enhance coverage across the eye.^37^^–^^42^ Third, we added a human pan-body reference small conditional RNA (scRNA) dataset to allow for non-ocular comparisons.^43^ Fourth, we used an in silico method to remove background gene contamination, as we noticed persistent rhodopsin expression in many non-photoreceptors cells (Supplementary Fig. S4). Fifth, we increased the stringency of the cell retention cutoff for detected unique transcripts per cell from 200 to 300. Sixth, we carried over and harmonized several common cell-type labels from the Tabula Muris project to more easily provide non–eye cell-type comparisons.^44^ As before, the scVI integration parameters of the pipeline were chosen rigorously by testing integration performance across a wide range of latent dimensions and number of highly variable genes in our scPOP framework^23^ (Supplementary Fig. S1).

Of the 931,538 ocular cells, only 465,281 had cell-type labels that we could recover from the publications or data deposits. To label most of the remaining cells, we used our xgboost approach from Swamy et al.,^23^ which we previously found performed well when compared to other cell-type transfer approaches. The overall accuracy is 96%, and the confusion matrix shows that the predicted cell type to published cell type recall is very high for most cell types (Supplementary Fig. S5). We also see that the cell-type labeling works well across the different species (Supplementary Table S1). The cell types with lower labeling performance tended to be ciliary, limbal, and corneal cells (which were often labeled as fibroblasts). There are fewer unique datasets for these cell types, and we expect that future runs with more data will improve this performance.

**Figure 1.**
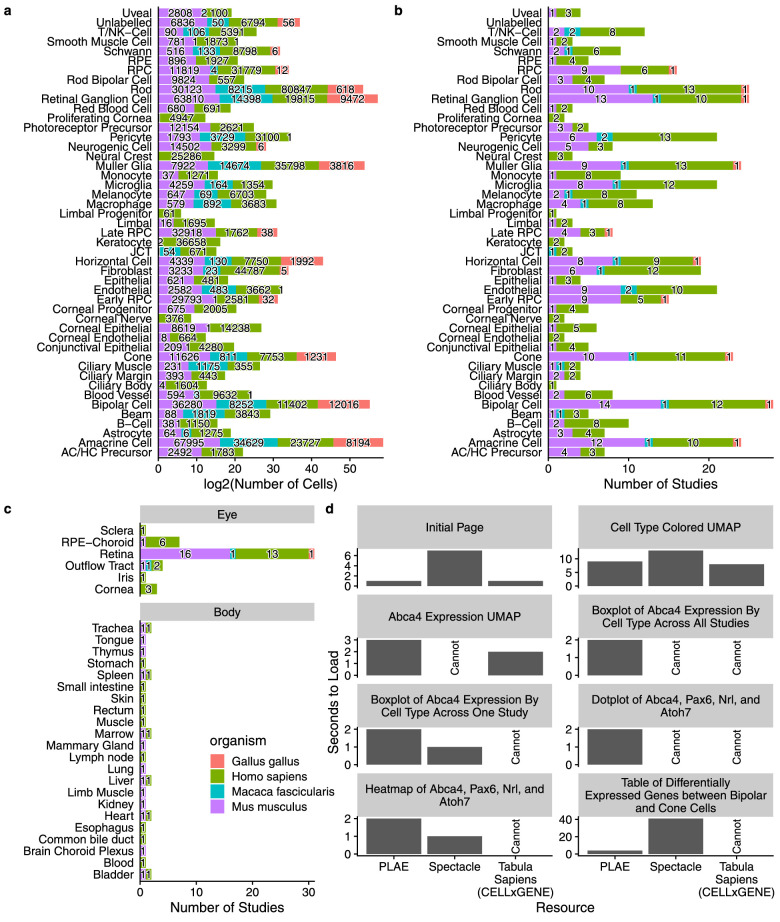
Distribution of tissues and cell types in scEiaD. (**a**) Number of cells for each ocular cell type. (**b**) Number of studies for each ocular predicted cell type (over 10 cells), colored by species. (**c**) Tabulation of the number of studies present across six ocular and 23 non-ocular tissues. (**d**) Web app timings (lower is better) for important functions compared among PLAE, Spectacle, and Tabula Sapiens (see Methods for more details).

After the dataset and quality control updates the scEiaD v2022-03-21 dataset now contains 44 studies, four species, 1,136,041 cells, and 60 curated cell types (Supplementary Fig. S6). The scEiaD database contains 23 non-ocular human and mouse tissues and six ocular tissues across human, chicken, macaque, and mouse (Fig. 1). This resource is only possible due to the publication of 35 single-cell resources from which scEiaD draws (Supplementary Table S2). To recognize these papers, we prominently feature each of these studies along with a direct link to their citation, where possible, on the loading page of plae.nei.nih.gov.

### PLAE Resource Contains Several Crucial Features Relative to Other Single Cell Resources

Several single-cell RNA-seq web-based resources contain ocular data (Table). The UCSC Cell Browser, Tabula Sapiens portal, PanglaoDB, and Protein Atlas are general-purpose, single-cell repositories that contain some ocular data. The PanglaoDB and UCSC resources have ingressed a large number of published single-cell datasets. As of late 2022, the UCSC Cell Browser had around 60,000 ocular single cell transcriptomes, and the PangloaDB had about 47,000. However, these resources do not curate and normalize metadata terms. This means that cell types have inconsistent naming, which makes cross-study comparisons very challenging. The Tabula Sapiens project is an effort to sequence all cell type transcriptomes in humans; although it contains only around 10,000 ocular cells it can be more readily cross-compared to non-ocular tissues/cells. A downside is that the ocular cell-type labels can be insufficient; for example, rods and cones are together labeled as photoreceptors. The Protein Atlas resource has used only one retina single-cell RNA-seq paper to provide information focused on cell-type label for gene queries (e.g., one can see whether a gene of interest is enriched in Cones). The use by the Protein Atlas of only one study is problematic, as it is unclear whether signal found is specific to the one dataset they use or is common across independent species and datasets.

**Table.** Comparison of PLAE With Other Web Resources That Contain Single-Cell Ocular Datasets as of March 15, 2021

Resource Name	Independent Datasets?	Harmonization?	Exportable?^a^	Organisms,^b^ *n*	Ocular and Non-Ocular Data?	Ocular Cell Count (1000s), *n*	URL
PLAE	Yes	Yes	Yes	4	Yes	1136	https://plae.nei.nih.gov
Spectacle	Yes	No	No	5	No	1500	https://singlecell-eye.org
UCSC Cell Browser	Yes	No	Yes	2	Yes	62	https://cells.ucsc.edu
Tabula Sapiens	No	N/A	Yes	1	Yes	10	https://tabula-sapiens-portal.ds.czbiohub.org
PanglaoDB	Yes	No	Yes	2	Yes	47	https://panglaodb.se
Protein Atlas	Yes^c^	No	No	1	Yes	5	https://www.proteinatlas.org/humanproteome/celltype

a Exportable means whether a resource allows bulk downloads of the count matrices and metadata.

b The Organisms column refers to the number of ocular species with ocular data.

c Although the Protein Atlas contains independent datasets, it has only one ocular study.

The Spectacle web resource is the closest comparison to PLAE, as it is a ocular focused resource that has collated a large number of ocular single-cell RNA-seq resources (both organoid and tissue based datasets total well over a million cells) and made them available on a web app. PLAE is distinguished from these resources by hand curation of the cell- and sample-level metadata, reprocessing the raw sequence data in a consistent framework, batch correcting the independent studies, and, crucially, providing full availability of the underlying data for outside usage.

Although PLAE and Spectacle (https://singlecell-eye.org) both contain large numbers of ocular-related scRNA datasets, they are structured differently.^22^ PLAE is built around the scEiaD database, which uses a multi-stage pipeline to build and identify a high performing scVI-based single-cell RNA eye model that is used to integrate all datasets together.^23^ This allows for gene queries to be constructed and analyzed across studies. In contrast, Spectacle, as of August 2022, was a compilation of study-level datasets, which makes queries across studies impossible. Spectacle does not contain any non-ocular datasets, and the cell-type labels have not been curated or harmonized among studies (for example different studies can use MG, Mueller, Müller Glia, or other variations to refer to the same cell type). Spectacle does contain more cells than PLAE because it also contains organoid and perturbed studies. Finally, unlike the PLAE resource, Spectacle does not make their datasets available for download in any form.

### Extensive Optimization Makes the PLAE Web App Highly Responsive Despite Containing Huge Amounts of Data

There are two interwoven reasons why the PLAE app is quick, despite the huge amount of data it contains. First, the data structure of scEiaD is a sqlite database with pre-calculated tests stored in tables. A sqlite database and its indexing functionality allows large amounts of data to be stored for quick retrieval with minimal loading time and memory usage. The major downside is that this approach requires a large amount of disk storage, which can make local (on a personal computer) usage difficult; the scEiaD v2022-03-22 sqlite database is about 368 GB. Second, the visualization code in PLAE (e.g., the UMAP view) pulls from the custom database directly and uses a highly optimized plotting system (scattermore) to reduce drawing time. In contrast, starting from the loading page of Spectacle, it takes approximately 11 seconds to plot the cell type metadata for a several thousand cells across a single study (tested on August 26, 2022). Within the same time, PLAE can plot the cell type labels for over a million cells across 37 publications (Supplementary Fig. S6d). The speed of the PLAE app is also comparable to the CELLxGENE browser serving the Tabula Sapiens resource while providing additional functionality.

### Rich Web-Available Visualizations

**Figure 2.**
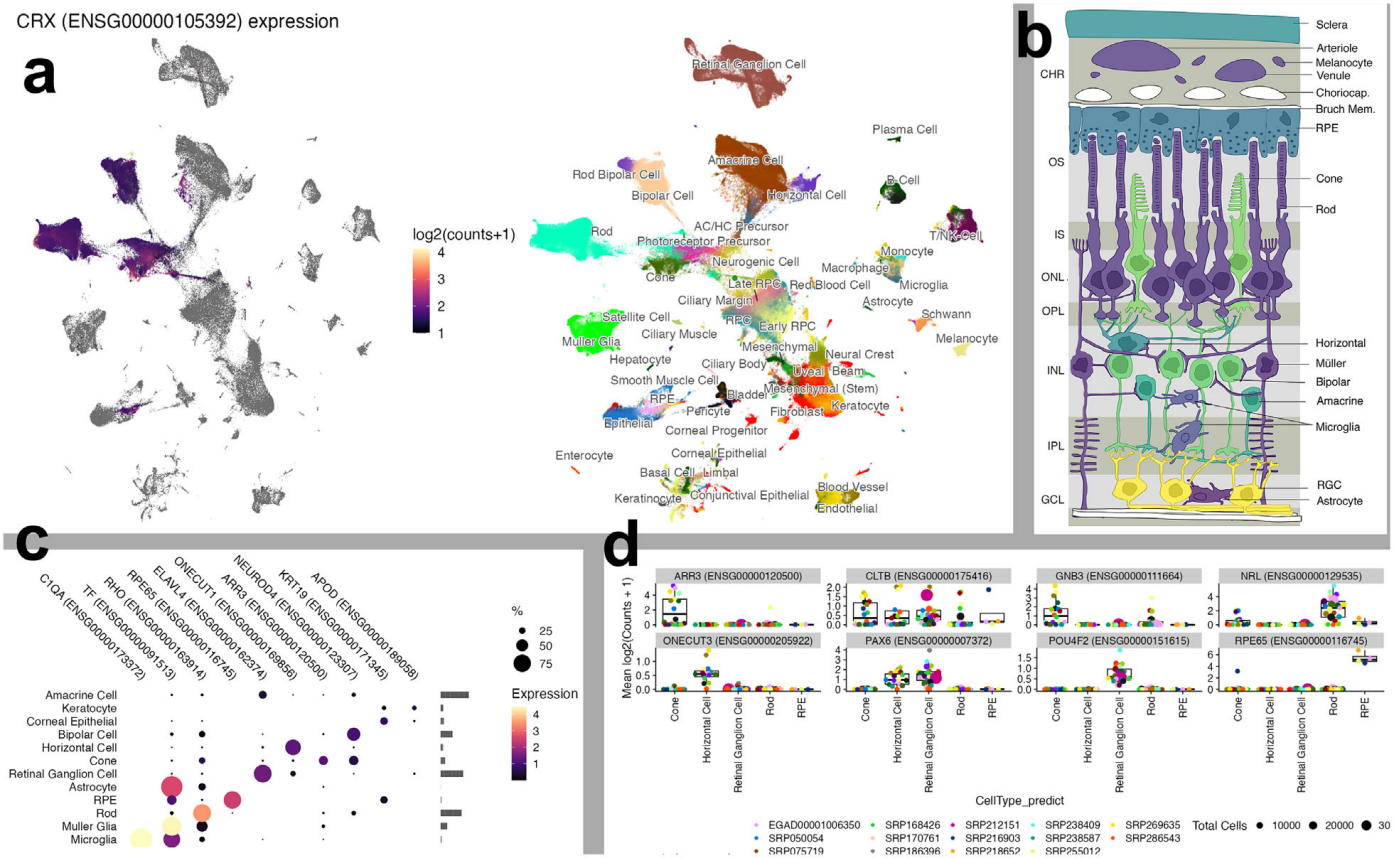
Screenshots from PLAE app. (**a**) UMAP view showing *Crx* expression with cell-type labels. (**b**) In silico in situ view of *Cltb*. (**c**) Dot plot demonstrating efficient display of gene expression across many cell types. (**d**) Box plot of eight genes, with each overlaid point representing an individual study.

PLAE has several ways to visualize the data, one being the two-dimensional (2D) UMAP view, which, side-by-side, shows gene–cell expression and metadata–cell values. Thus, a user can see *Crx* expression across over a million cells and dozens of studies and separately see cell-type labels for those same million cells. The 2D UMAP view also has a zoom function, so a user can focus on a subset of cells by clicking and dragging to set a zoom in region. Clicking on the plot will show metadata for the five nearest cells to the click, which is useful to seeing what kind of cells are present in a small area (Fig. 2a).

For users interested in seeing how expression of a gene relates to study or other covariates, the Expression Plot view displays expression by cell type, predicted cell type, or cluster. The values can then be split and colored by multiple covariates like study or organism. This allows a user to see whether a gene pattern is consistent across studies or species. We also offer a Dot Plot view, which can display a large number of genes in a space efficient manner. This allows users to see whether a set of genes has related (or not) expression patterns across multiple cell types (Figs. 2b, 2c).

Although scEiaD does not have spatial scRNA-seq datasets as of 2023, it does leverage existing knowledge of the structure of the retina to show in silico visualization of genes by cell type/layer in a cartoon cross-section of the retina. The structure of the cartoon retina is based on the human retina. This unique visualization displays the major cell types of the retina and the RPE/choroid layer behind it. The cell types are colored by the relative amounts of expression in the user-selected gene. Like all visualizations in PLAE, the user can use powerful filtering options to display only data from certain metadata characteristics, such as species or publication (Fig. 2d).

We have also added a heatmap visualization, which summarizes the relative expression differences between user-selected cell types or clusters across human, macaque, and mouse. This plot differs from the Dot Plot view, as the expression values are relative instead of absolute. That is to say that, although the Dot Plot view is proportionate to counts, the Heatmap view is showing the difference in expression between a given cell type (or cluster) and all other cells as calculated by DESeq2. This view is potentially useful if a user is especially interested in gene expression patterns across a variety of cell types. One downside is that, because we currently only have one *Gallus gallus* dataset, we cannot use that data in this visualization because we cannot calculate a meaningful differential expression with only one sample.

### Well-Supported Ocular Cell Types Identify High-Confidence Cell-Type Markers

Cell-type marker genes can be proposed on the basis of existing knowledge about their function and on high expression differences when comparing to other cell types. We can further narrow down the list of markers by using the high diversity of studies and organisms in scEiaD to propose a set of community-supported cell-type markers. We first identified a set of well-supported cell types, which we defined as cell types that are detected in two or more independent studies across both human and mouse (Supplementary Table S4). These cell types were then assessed to identify differentially expressed genes that met the following criteria across human, mouse, and macaque: (1) adjusted *P* value (padj) less than 1 × 10^–4^ in two or more species, (2) mean log2 fold change greater than 2, and (3) mean padj less than 1 × 10^–5^. A final filter was applied to remove genes as candidates if they were differentially expressed (using the criteria above) in more than three different cell types. This left us with 2790 gene–cell type markers, a substantial reduction from the 33,927 we had initially. We provide a table of these genes in the Supplementary Material (consistently_differentially_expressed_genes.csv.gz).

**Figure 3.**
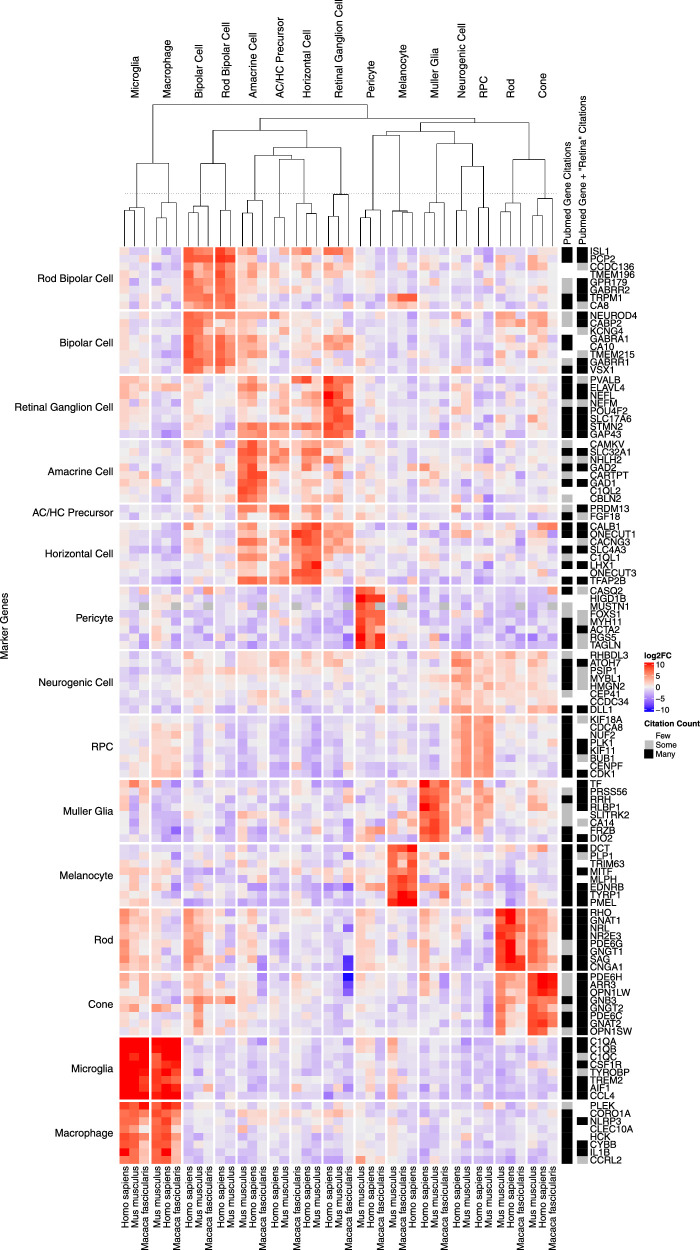
*Rows* are genes, *columns* are cell types and organisms. *Rows* are split by what cell type the gene is a marker for. *Columns* are split by the cell types. More *intense red* is a higher log2 fold change of the gene–cell type relative to all other cell types, and *blue* is more negative. Relative numbers of citations are shown for the gene or gene “AND Retina” when searching against PubMed compared to 1000 randomly chosen genes.

To visualize these top markers we selected up to eight genes (ordered by the mean log2 fold change across organisms) per well-supported cell type for each and plotted the log2 fold change of the gene expression relative to all other cell types (Fig. 3). We could see that human, mouse, and macaque have similar expression patterning of the proposed markers across the well-supported cell types. The cell types are arranged by the columns by how related the expression patterns are. We can see how the differentiated cell types of the retina are grouped together apart from the Müller glia, which are near the retinal progenitor cells (RPCs) from which they are derived.

To assess whether this marker selection was identifying known or potentially novel marker genes we ran several sets of queries against PubMed. We searched for the gene name or the gene name plus “AND Retina” across all well-supported genes against the PubMed database, counting how many citations were returned. In contrast, we also selected 1000 random genes that were not considered differentially expressed and ran the same PubMed searches. We found a substantial enrichment of citations for our well-supported differentially expressed genes (*t*-test, *P* < 6.7 × 10^–98^ and *P* < 2.4 × 10^–12^, respectively) for the gene and gene “AND Retina” PubMed searches when compared against the 1000 random genes. To display the results of this search for each individual gene, we showed visually whether the number of citations returned was less or equal than the median citation count for the random gene set (“few”), more than the median (“some”), or more than the mean (“many”). We see that nearly all genes in Figure 3 reflected existing knowledge.

Genes with relatively few citations can be considered as novel markers. We see, for example, that *Onecut3* appears to well separate the horizontal cells and has few PubMed citations. To assess whether there are any other good candidates with few citations, we produced another heatmap (Supplementary Fig. S7). Most of these genes are differentially expressed across more than one cell type. We hand selected *C1ql2* and *Cartpt* (amacrine), *Frzb* and *Slitrk2* (Müller glia), and *Onecut3* (horizontal) and plotted these five genes in the Box Plot view to confirm specificity across the full scEiaD database (Supplementary Fig. S8). All five seem to be specific to a cell type, except for *FRZB*, which also is expressed in the RPE.

Other strategies for ranking genes are possible. We show in Supplementary Figure S9b and S9c how one can directly use our powerful web app to select candidate bipolar cell differentially expressed genes from the differential testing table and then quickly display the expression pattern of those genes in a compact heatmap visualization. The wealth of visualization options in plae allow for quick prototyping and hypothesis testing of gene expression patterning across many cells and studies.

### Tease Apart the Similar Neurogenic and Retinal Progenitor Cell Types

**Figure 4.**
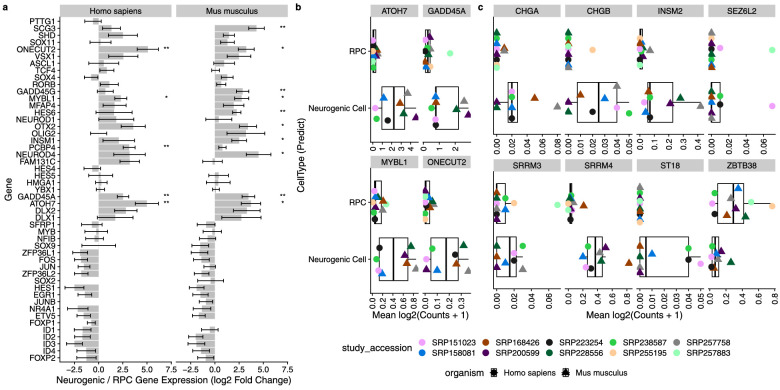
(**a**) Genes proposed by Lu et al.^45^ as being significantly differentially expressed during the RPC to neurogenic cell differentiation in human. We plotted the differential expression (log2 fold change) directly between RPCs and neurogenic cells. The standard error (calculated by DESeq2) is plotted. *Genes with padj < 0.1. **Genes with padj < 0.01. (**b**) Genes from Lu et al.^45^ that we replicated with the scEiaD data. (**c**) Novel genes that we propose are differentially expressed between RPCs and neurogenic cells.

Although the well supported markers we propose clearly define most of the cell types, we see that the neurogenic cells and RPCs have very similar expression patterns across the gene sets. These cell types are closely related, as the neurogenic cells are derived from the multi-potent RPC to later specify neural cell types. We first used the set of genes identified by Lu et al.^45^ in their study of human retinal differentiation to determine whether our scEiaD database retained the same trends (Fig. 4A). As expected, we observed several patterns. First, most of the gene expression changes between RPCs and neurogenic cells are matched between humans. We further found that these genes also showed the same trends in mouse datasets. Next, we observed that the direction of the changes largely matched that of Lu et al.^45^ (Supplementary Fig. S10). Finally, we saw that a small number of genes were confirmed in our database as consistently differentially expressed with a padj < 0.1.

**Figure 5.**
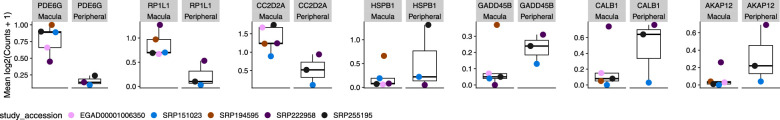
Plot of markers distinguishing human macula or peripheral punch-derived cones.

To determine whether we can propose any new genes that distinguish RPC and neurogenic states we used our DESeq2 differential expression contrast, which directly compares these two cell types. We find 88 human genes and 91 mouse genes that have an absolute log2 fold change greater than 2 and a padj < 0.05 (Supplementary Table S5). To identify candidate genes that are consistently differentially expressed at this transitional state in mouse and human, we applied logic similar to that we used in identifying well-supported marker genes across the retina cell types. We filtered to genes with a padj < 0.05 in both human and mouse and a mean log2 fold change greater than 2 or less than –1 (Figs. 4B, 4C). We found one gene, *ZBTB38*, that meets this criteria and drops in expression when comparing RPCs to neurogenic cells. We found 11 genes that increase in expression from RPCs to neurogenic cells, seven of which are not previously identified by Lu et al.^45^ (Fig. 4C). We replicated *ATOH7*, *ONECUT2*, *GADD45A*, and *MYBL1* from Lu et al.^45^ (Fig. 4B) as differentially expressed between neurogenic cells and RPCs. Furthermore, we show in Supplementary Figure S9a how this figure can be replicated directly in our web app.

### Our Pan-Study Macula Versus Peripheral Cone Test Replicates a Published Macaque Finding and Proposes Six New Markers

Although we have provided data in many forms via the web app at plae.nei.nih.gov, there are many more dissections of the data that can be performed. To facilitate custom testing with the scEiaD database, we provide, as an example, a human-specific macula versus peripheral cone differential testing analysis document in the Supplementary Material (pseudobulk_cone_region.freeze01.html). In our analysis document, we used the Seurat object containing the full scEiaD dataset we provide at plae.nei.nih.gov to build a custom pseudobulk counts matrix containing only cones from human studies that were taken in a region-specific manner. This left us with 26,678 cells across six studies. Yan et al.^2^ previously proposed *Prph2* and *Rs1* as markers to distinguish macula and peripherally located human cones, and Peng et al.^46^ proposed *Calb1* and *Gngt1* as cone region markers in macaque.

Our differential testing found that, although *Prph2*, *Rs1*, and *Gngt1* had expression differences matching that the findings that Yan et al.^2^ and Peng et al.^46^ published, they failed to replicate in our test with a *P* value being above 0.1 for all three. In contrast *Calb1* did replicate strongly with the sixth lowest padj (0.00721) across our entire test and a log2 fold change of 2.87. We found six more genes with a padj < 0.01: *Pde6g*, *Akap12*, *Hspb1*, *Gadd45b*, *Cc2d2a*, and *RP1l1* (Fig. 5, Supplementary Table S6). None of these candidate cone region marker genes displays regionally different expression in rods (Supplementary Fig. S11).

### Limitations of scEiaD

The longer term goal is to continue adding datasets to the scEiaD at plae.nei.nih.gov until we have three or more independent datasets per species and most ocular cell types. Earlier we proposed well-supported cell types. Right now, most of these well-supported cell types are in the retina. The RPE behind the retina, the cornea and lens in front, and the outflow tract and supporting musculature still could use more independent studies. Many common human diseases underlie the RPE (e.g., age-related macular degeneration) and the cornea (e.g., keratoconus), and these tissues will remain a focus for the next update of our database. Although we have curated a large number of cell types across the eye, many of these cell types have “sub”-cell types. For example the human retina has three types of cones that are optimized for short, medium, and long wavelengths. More dramatically, Yan et al.^47^ proposed over 60 different mouse amacrine cell types. We have found it fairly straightforward to use machine learning to transfer the broad (e.g., rod, cone, amacrine) cell-type labels across all datasets, but we have found it very challenging to transfer the sub-cell-type labels. This is largely because, although we have a high diversity of studies for the broad cell types, this is not true for most of the sub-cell types. We believe more datasets that carefully dissect the sub-cell types may be necessary to confidently propose the sub-cell types of the retina. Finally, although our scVI-based model can theoretically be used by an outside group to transfer their internal data into the scEiaD latent space, it is very challenging to implement as the software landscape is still rapidly changing, and it is very difficult to architect consistent outputs over time with regularly changing software versions. We are investigating simpler methods to enable transfer of knowledge from scEiaD to outside data.

## Conclusions

### High Feature Web App Provides Quick Access to Over One Million Transcriptomes Across 44 Datasets

We have improved on the scEiaD dataset we published in Swamy et al.^23^ by adding another species (*Gallus gallus*), new ocular tissues, and a human non-ocular reference set—in total, 10 new single-cell datasets. We restructured the data into a custom database to enable our custom-built high-performance web app to quickly access the data. This democratizes access to over a million cells across 44 studies to anyone with a web browser. We recognize that, although we have built a powerful visualization platform, we cannot anticipate all uses; for those researchers who wish to make custom queries of the data, we provide the data underlying PLAE in a wide variety of formats, including anndata and Seurat objects, for each study, count tables, cell-level metadata, and the DESeq2-based differential testing across three species. We provide a short example of how a novel query could work by running a custom differential test between macula and peripheral derived human cones and demonstrate how this can both validate existing proposed genes and suggest new ones.

### Diversity of Studies Enables Reproduction and Extension of Cell Type Gene Expression Findings

A great many groups have proposed cell atlases. These range from efforts focusing on a subset of cells from a single layer of a tissue^48^ to a huge consortium encompassing multiple organs.^18^ We propose a complementary approach, wherein we use the research data from the community at large to create a meta-atlas. This strategy enables a small group to produce an atlas that rivals the size from massive multi-group consortiums. Furthermore, the inherent variability between the data from independent groups can be used as a feature to obtain insight into whether a gene cell-type signal is stable across an entire community's datasets. We use this feature to demonstrate how the high study diversity in scEiaD can be used to validate proposed RPC to neurogenic cell gene expression changes in an R analysis document. Our community-powered, single-cell, ocular meta-atlas enables the entire ocular community to use the wealth of research it has generated.

## Supplementary Material

Supplement 1

Supplement 2

Supplement 3

Supplement 4

## References

[1] Masland RH. Cell Populations of the retina: the proctor lecture. *Invest Ophthalmol Vis Sci*. 2011; 52(7): 4581–4591. 21712414 10.1167/iovs.10-7083 PMC3175966

[2] Yan W, Peng YR, van Zyl T, et al. Cell atlas of the human fovea and peripheral retina. *Sci Rep*. 2020; 10(1): 9802. 32555229 10.1038/s41598-020-66092-9 PMC7299956

[3] Hagstrom SA, Neitz M, Neitz J. Cone pigment gene expression in individual photoreceptors and the chromatic topography of the retina. *J Opt Soc Am A Opt Image Sci Vis*. 2000; 17(3): 527–537. 10708034 10.1364/josaa.17.000527

[4] Jabs R, Guenther E, Marquordt K, Wheeler-Schilling TH. Evidence for P2X(3), P2X(4), P2X(5) but not for P2X(7) containing purinergic receptors in Müller cells of the rat retina. *Brain Res Mol Brain Res*. 2000; 76(2): 205–210. 10762695 10.1016/s0169-328x(99)00339-3

[5] Li H, Gaughwin P, Li N, He S. Localization of dopamine D1-receptor to A-type horizontal cells in the rabbit retina by single cell RT-PCR. *Neurosci Lett*. 2004; 355(1-2): 146–148. 14729255 10.1016/j.neulet.2003.10.042

[6] Lindqvist N, Vidal-Sanz M, Hallböök F. Single cell RT-PCR analysis of tyrosine kinase receptor expression in adult rat retinal ganglion cells isolated by retinal sandwiching. *Brain Res Mol Brain Res*. 2002; 10(2): 75–83. 10.1016/s1385-299x(02)00184-8 12431706

[7] Reid SNM, Yamashita C, Farber DB. Retinoschisin, a photoreceptor-secreted protein, and its interaction with bipolar and Müller cells. *J Neurosci*. 2003; 23(14): 6030–6040. 12853421 10.1523/JNEUROSCI.23-14-06030.2003 PMC6740352

[8] Cherry TJ, Trimarchi JM, Stadler MB, Cepko CL. Development and diversification of retinal amacrine interneurons at single cell resolution. *Proc Natl Acad Sci USA*. 2009; 106(23): 9495–9500. 19470466 10.1073/pnas.0903264106 PMC2686638

[9] Gustincich S, Contini M, Gariboldi M, et al. Gene discovery in genetically labeled single dopaminergic neurons of the retina. *Proc Natl Acad Sci USA*. 2004; 101(14): 5069–5074. 15047890 10.1073/pnas.0400913101 PMC387375

[10] Kim DS, Ross SE, Trimarchi JM, Aach J, Greenberg ME, Cepko CL. Identification of molecular markers of bipolar cells in the murine retina. *J Comp Neurol*. 2008; 507(5): 1795–1810. 18260140 10.1002/cne.21639 PMC2665264

[11] Roesch K, Jadhav AP, Trimarchi JM, et al. The transcriptome of retinal Müller glial cells. *J Comp Neurol*. 2008; 509(2): 225–238. 18465787 10.1002/cne.21730 PMC2665263

[12] Trimarchi JM, Stadler MB, Cepko CL. Individual retinal progenitor cells display extensive heterogeneity of gene expression. *PLoS One*. 2008; 3(2): e1588. 18270576 10.1371/journal.pone.0001588 PMC2220035

[13] Trimarchi JM, Stadler MB, Roska B, et al. Molecular heterogeneity of developing retinal ganglion and amacrine cells revealed through single cell gene expression profiling. *J Comp Neurol*. 2007; 502(6): 1047–1065. 17444492 10.1002/cne.21368

[14] Wahlin KJ, Lim L, Grice EA, Campochiaro PA, Zack DJ, Adler R. A method for analysis of gene expression in isolated mouse photoreceptor and Müller cells. *Mol Vis*. 2004; 10: 366–375. 15205663

[15] Klein AM, Mazutis L, Akartuna I, et al. Droplet barcoding for single-cell transcriptomics applied to embryonic stem cells. *Cell*. 2015; 161(5): 1187–1201. 26000487 10.1016/j.cell.2015.04.044 PMC4441768

[16] Macosko EZ, Basu A, Satija R, et al. Highly parallel genome-wide expression profiling of individual cells using nanoliter droplets. *Cell*. 2015; 161(5): 1202–1214. 26000488 10.1016/j.cell.2015.05.002 PMC4481139

[17] Nieuwenhuis TO, Yang SY, Verma RX, et al. Consistent RNA sequencing contamination in GTEx and other data sets. *Nat Commun*. 2020; 11(1): 1933. 32321923 10.1038/s41467-020-15821-9 PMC7176728

[18] Tabula Sapiens Consortium, Quake SR. A multiple-organ, single-cell transcriptomic atlas of humans. Science. 2022; 376(6594): eabl4896. 35549404 10.1126/science.abl4896 PMC9812260

[19] Franzén O, Gan LM, Björkegren JLM, Panglao DB. A web server for exploration of mouse and human single-cell RNA sequencing data. *Database (Oxford)*. 2019; 2019: baz046. 30951143 10.1093/database/baz046 PMC6450036

[20] Karlsson M, Zhang C, Méar L, et al. A single-cell type transcriptomics map of human tissues. *Sci Adv*. 2021; 7(31): eabh2169. 34321199 10.1126/sciadv.abh2169 PMC8318366

[21] Speir ML, Bhaduri A, Markov NS, et al. UCSC cell browser: visualize your single-cell data. *Bioinformatics*. 2021; 37(23): 4578–4580. 34244710 10.1093/bioinformatics/btab503 PMC8652023

[22] Voigt AP, Whitmore SS, Lessing ND, et al. Spectacle: an interactive resource for ocular single-cell RNA sequencing data analysis. *Exp Eye Res*. 2020; 200: 108204. 32910939 10.1016/j.exer.2020.108204 PMC7655596

[23] Swamy VS, Fufa TD, Hufnagel RB, McGaughey DM. Building the mega single-cell transcriptome ocular meta-atlas. *GigaScience*. 2021; 10(10): giab061. 34651173 10.1093/gigascience/giab061 PMC8514335

[24] Mölder F, Jablonski KP, Letcher B, et al. Sustainable data analysis with Snakemake. *F1000Res*. 2021; 10: 33. 34035898 10.12688/f1000research.29032.1 PMC8114187

[25] Melsted P, Ntranos V, Pachter L. The barcode, UMI, set format and BUStools. *Bioinformatics*. 2019; 35(21): 4472–4473. 31073610 10.1093/bioinformatics/btz279

[26] Melsted P, Booeshaghi AS, Gao F, et al. Modular, efficient and constant-memory single-cell RNA-seq preprocessing. *Nat Biotechnol*. 2021; 39(21): 813–818. 33795888 10.1038/s41587-021-00870-2

[27] Lun ATL, Riesenfeld S, Andrews T, et al. EmptyDrops: distinguishing cells from empty droplets in droplet-based single-cell RNA sequencing data. *Genome Biol*. 2019; 20(1): 63. 30902100 10.1186/s13059-019-1662-y PMC6431044

[28] Wolock SL, Lopez R, Klein AM. Scrublet: computational identification of cell doublets in single-cell transcriptomic data. *Cell Syst*. 2019; 8(4): 281–291.e9. 30954476 10.1016/j.cels.2018.11.005 PMC6625319

[29] Lun ATL, McCarthy DJ, Marioni JC. A step-by-step workflow for low-level analysis of single-cell RNA-seq data with Bioconductor. *F1000Res*. 2016; 5: 2122. 27909575 10.12688/f1000research.9501.1 PMC5112579

[30] Yang S, Corbett SE, Koga Y, et al. Decontamination of ambient RNA in single-cell RNA-seq with DecontX. *Genome Biol*. 2020; 21(1): 57. 32138770 10.1186/s13059-020-1950-6 PMC7059395

[31] Xu C, Lopez R, Mehlman E, Regier J, Jordan MI, Yosef N. Probabilistic harmonization and annotation of single-cell transcriptomics data with deep generative models. *Mol Syst Biol*. 2021; 17(1): e9620. 33491336 10.15252/msb.20209620 PMC7829634

[32] McCarthy DJ, Campbell KR, Lun ATL, Wills QF. Scater: pre-processing, quality control, normalization and visualization of single-cell RNA-seq data in R. *Bioinformatics*. 2017; 33(8): 1179–1186. 28088763 10.1093/bioinformatics/btw777 PMC5408845

[33] Squair JW, Gautier M, Kathe C, et al. Confronting false discoveries in single-cell differential expression. *Nat Commun*. 2021; 12(1): 5692. 34584091 10.1038/s41467-021-25960-2 PMC8479118

[34] Love MI, Huber W, Anders S. Moderated estimation of fold change and dispersion for RNA-seq data with DESeq2. *Genome Biol*. 2014; 15(12): 550. 25516281 10.1186/s13059-014-0550-8 PMC4302049

[35] Swamy V, McGaughey D. Eye in a disk: eyeIntegration human pan-eye and body transcriptome database version 1.0. *Invest Ophthalmol Vis Sci*. 2019; 60(8): 3236–3246. 31343654 10.1167/iovs.19-27106 PMC6660187

[36] Yamagata M, Yan W, Sanes JR. A cell atlas of the chick retina based on single-cell transcriptomics. *eLife*. 2021; 10: e63907. 33393903 10.7554/eLife.63907 PMC7837701

[37] Collin J, Queen R, Zerti D, et al. A single cell atlas of human cornea that defines its development, limbal progenitor cells and their interactions with the immune cells. *Ocul Surf*. 2021; 21: 279–298. 33865984 10.1016/j.jtos.2021.03.010 PMC8343164

[38] Gautam P, Hamashima K, Chen Y, et al. Multi-species single-cell transcriptomic analysis of ocular compartment regulons. *Nat Commun*. 2021; 12(1): 5675. 34584087 10.1038/s41467-021-25968-8 PMC8478974

[39] Dani N, Herbst RH, McCabe C, et al. A cellular and spatial map of the choroid plexus across brain ventricles and ages. *Cell*. 2021; 184(11): 3056–3074.e21. 33932339 10.1016/j.cell.2021.04.003 PMC8214809

[40] Ligocki AJ, Fury W, Gutierrez C, et al. Molecular characteristics and spatial distribution of adult human corneal cell subtypes. *Sci Rep*. 2021; 11(1): 16323. 34381080 10.1038/s41598-021-94933-8 PMC8357950

[41] Patel G, Fury W, Yang H, et al. Molecular taxonomy of human ocular outflow tissues defined by single-cell transcriptomics. *Proc Natl Acad Sci USA*. 2020; 117(23): 12856–12867. 32439707 10.1073/pnas.2001896117 PMC7293718

[42] van Zyl T, Yan W, McAdams A, et al. Cell atlas of aqueous humor outflow pathways in eyes of humans and four model species provides insight into glaucoma pathogenesis. *Proc Natl Acad Sci USA*. 2020; 117(19): 10339–10349. 32341164 10.1073/pnas.2001250117 PMC7229661

[43] He S, Wang LH, Liu Y, et al. Single-cell transcriptome profiling of an adult human cell atlas of 15 major organs. *Genome Biol*. 2020; 21(1): 294. 33287869 10.1186/s13059-020-02210-0 PMC7720616

[44] Single-cell transcriptomics of 20 mouse organs creates a Tabula Muris. *Nature*. 2018; 462(7727): 367–372. 10.1038/s41586-018-0590-4 PMC6642641 30283141

[45] Lu Y, Shiau F, Yi W, et al. Single-cell analysis of human retina identifies evolutionarily conserved and species-specific mechanisms controlling development. *Dev Cell*. 2020; 53(4): 473–491.e9. 32386599 10.1016/j.devcel.2020.04.009 PMC8015270

[46] Peng YR, Shekhar K, Yan W, et al. Molecular classification and comparative taxonomics of foveal and peripheral cells in primate retina. *Cell*. 2019; 176(5): 1222–1237.e22. 30712875 10.1016/j.cell.2019.01.004 PMC6424338

[47] Yan W, Laboulaye MA, Tran NM, Whitney IE, Benhar I, Sanes JR. Mouse retinal cell atlas: molecular identification of over sixty amacrine cell types. *J Neurosci*. 2020; 40(27): 5177–5195. 32457074 10.1523/JNEUROSCI.0471-20.2020 PMC7329304

[48] Tran NM, Shekhar K, Whitney IE, et al. Single-cell profiles of retinal ganglion cells differing in resilience to injury reveal neuroprotective genes. *Neuron*. 2019; 104(6): 1039–1055.e12. 31784286 10.1016/j.neuron.2019.11.006 PMC6923571

[49] Clark BS, Stein-O'Brien GL, Shiau F, et al. Single-cell RNA-seq analysis of retinal development identifies NFI factors as regulating mitotic exit and late-born cell specification. *Neuron.* 2019; 102(6): 1111–1126.e5. 31128945 10.1016/j.neuron.2019.04.010 PMC6768831

