# Supplementary material for: PLAE Web App Enables Powerful Searching and Multiple Visualizations Across One Million Unified Single-Cell Ocular Transcriptomes
Source: Transl Vis Sci Technol. 2023 Sep 25;12(9):18. doi: 10.1167/tvst.12.9.18 (PMC10578359; doi:10.1167/tvst.12.9.18)
Supplement: Supplement 1 [file tvst-12-9-18_s001.pdf]

Supplementary Figures and Tables

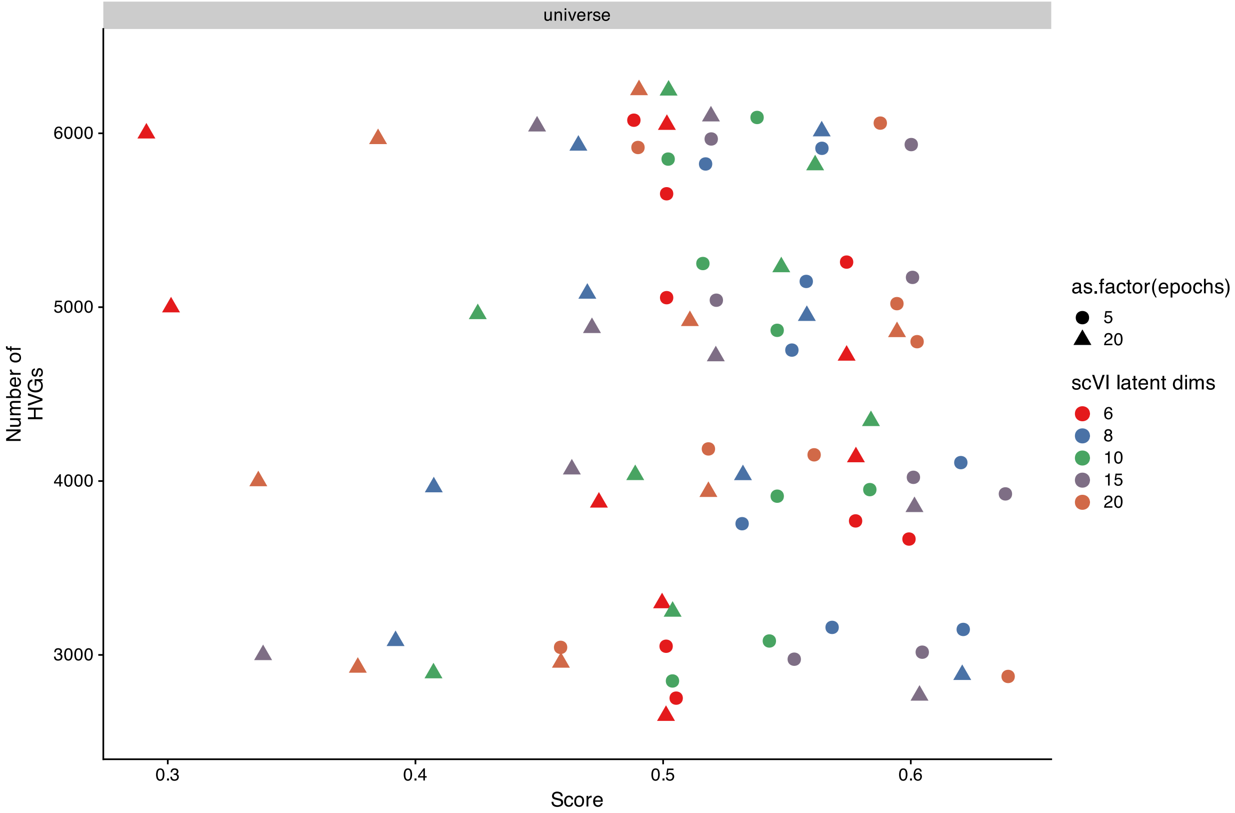

Supplemental Figure 1: scPOP based performance scoring (higher is better) across different numbers of highly variable genes, epochs of scANVI used, and number of outputted latent dimensions.

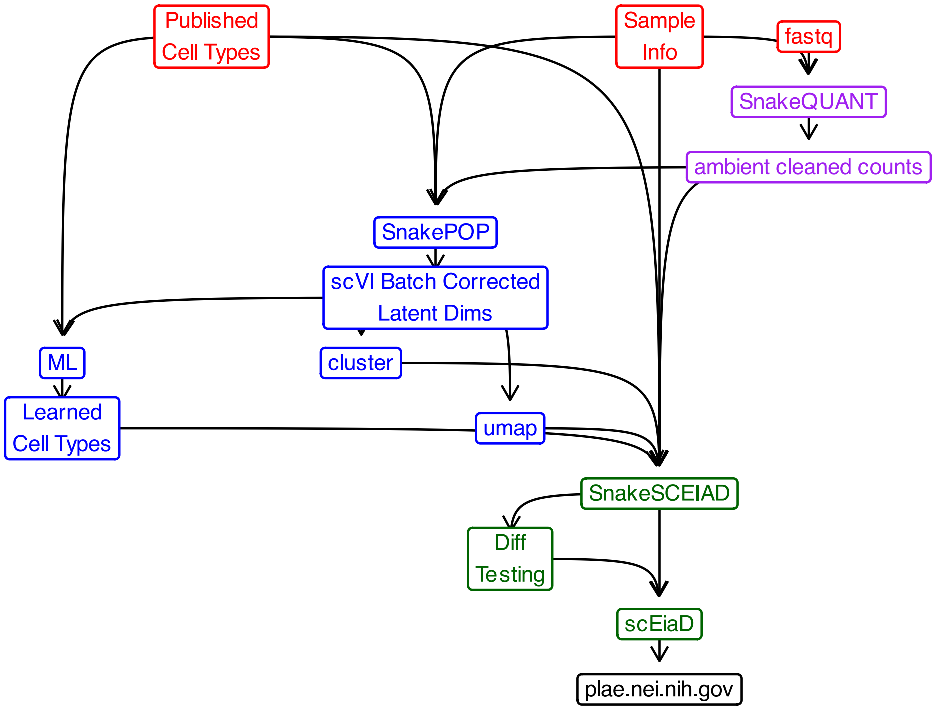

Supplemental Figure 2: Data flow overview from published cell types, sample information, raw fastq counts to produce the sqlite database used in plae.nei.nih.gov

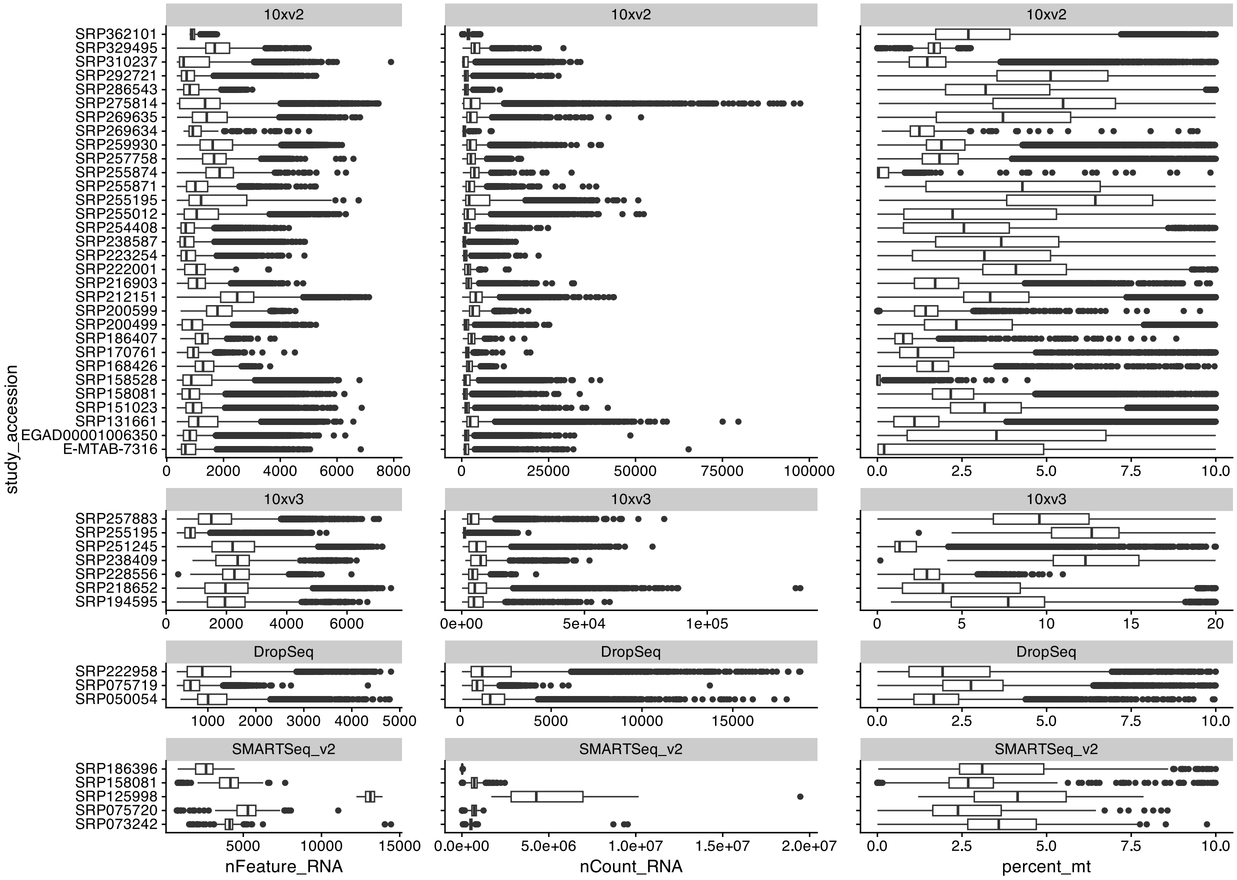

Supplemental Figure 3: Study level box plot showing unique genes identified, total gene counts, and percent mitochondrial reads.

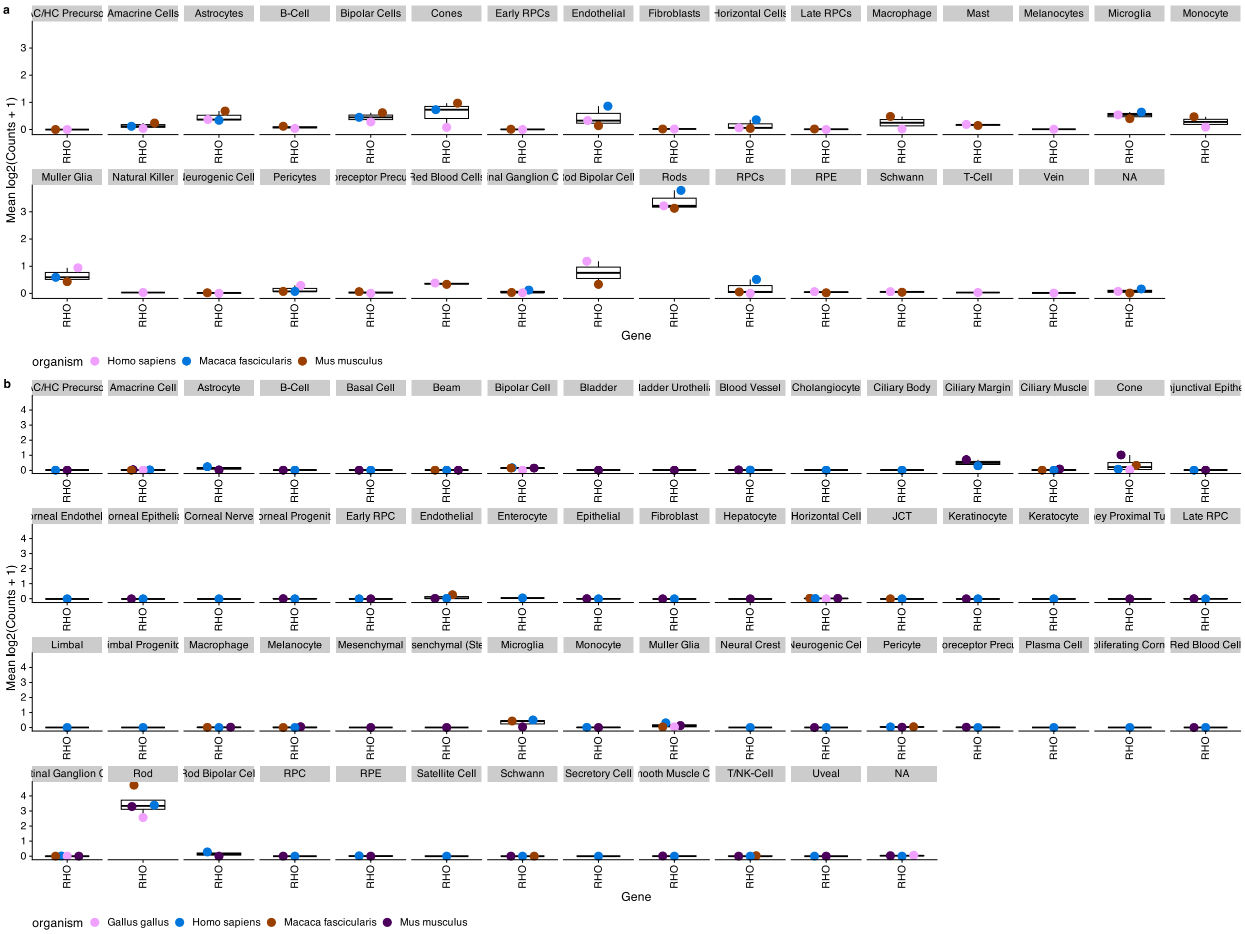

Supplemental Figure 4: DecontX in silico ambient RNA contamination tool substantially removes Rhodopsin expression in non-rod cells while retaining high expression rods. First shown (a) is Rhodopsin expression across the scEiaD v0 dataset, without DecontX optimization. Note Rhodopsin expression is noticeable in many cell types beyond the rods. Next (b) is the scEiaD v1 dataset with DecontX optimization. Rhodopsin is nearly exclusively expressed in labelled rod cells. Despite the computational ambient RNA removal, Rhodopsin expression remains high in the Rods.

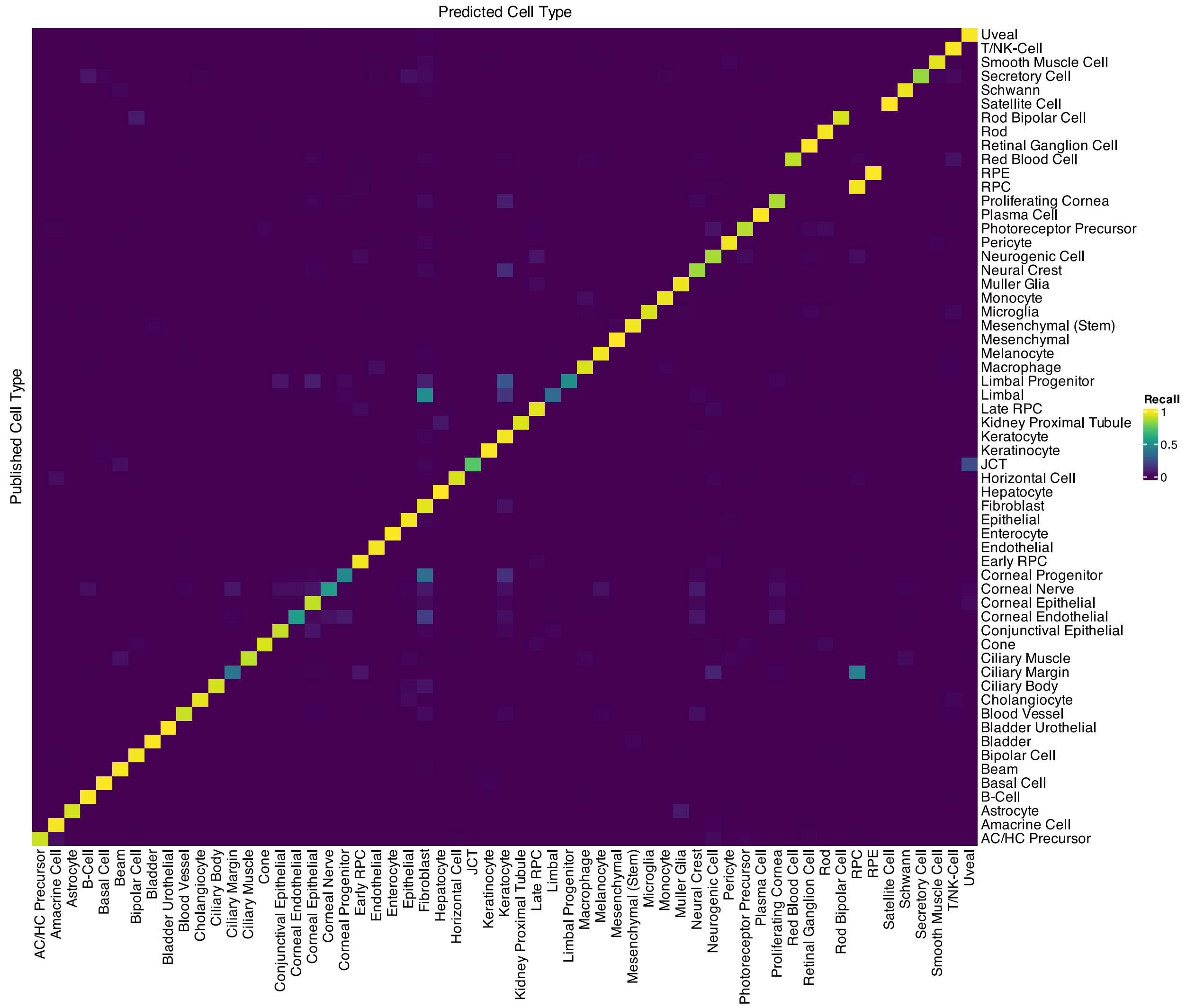

Supplemental Figure 5: Confusion matrix of cell type label consistency between predicted cell types (columns) and published cell types (rows).

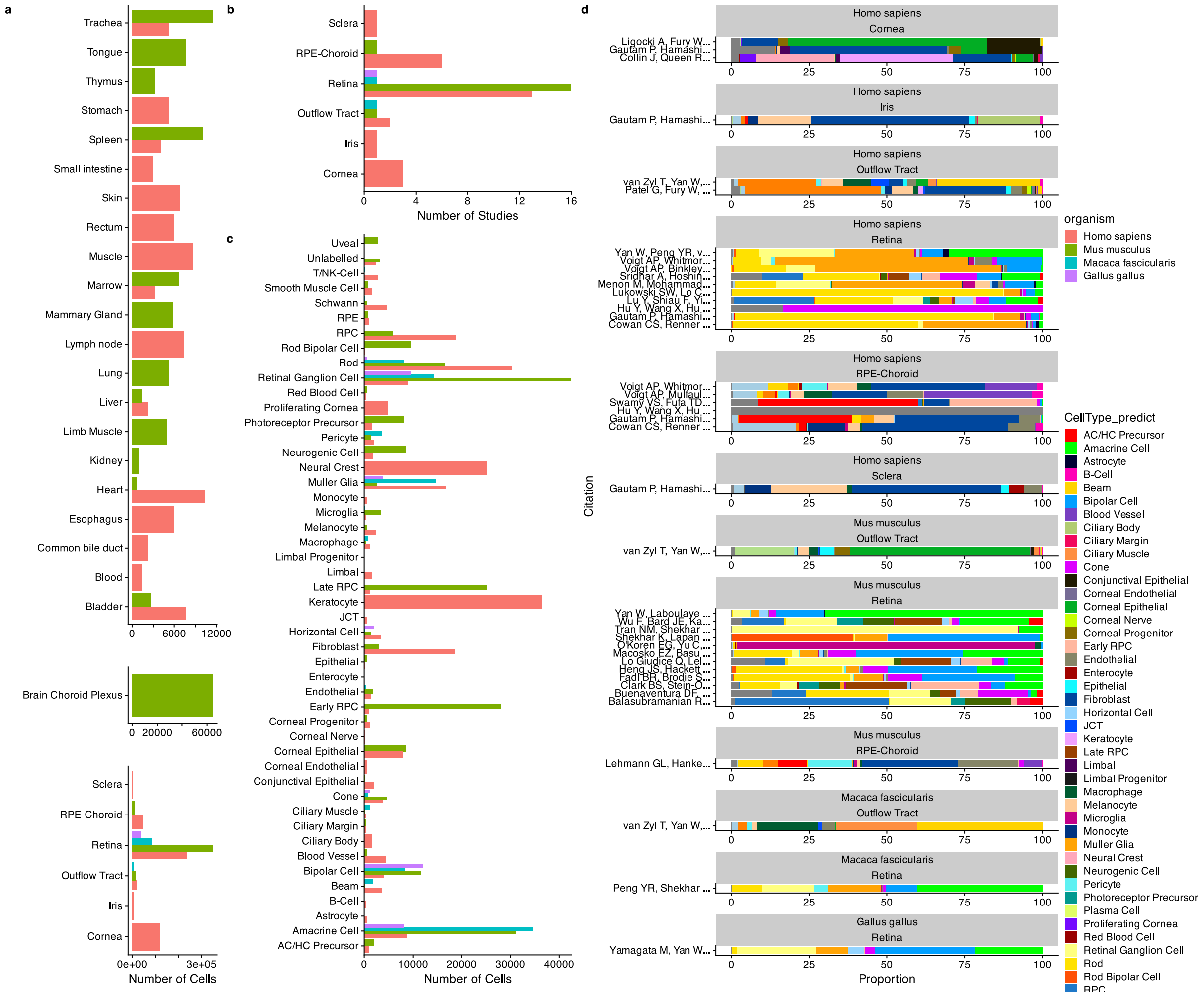

Supplemental Figure 6: Distribution of tissues and cell types in scEiaD. A. tabulation of the number of cells present across 6 ocular and 23 non-ocular tissues, B. Number of studies for each ocular tissue. C. Number of cells present across 53 curated ocular-derived cell types. D. Proportion of cell types (predicted) across each study (split by species and tissue).

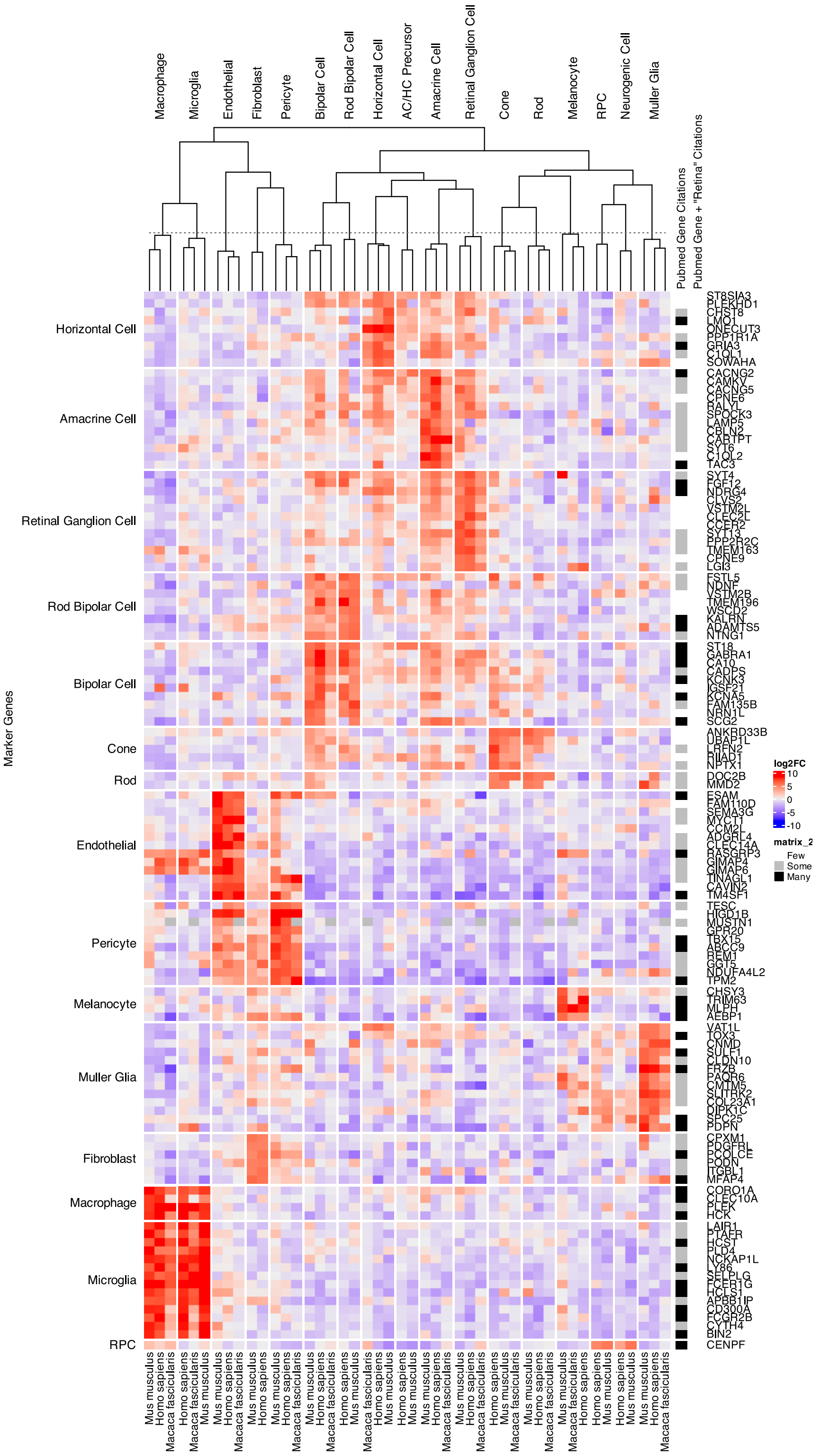

Supplemental Figure 7: Well supported genes with a mean log2FC > 5 and zero pubmed citation hits for [gene] AND Retina search.

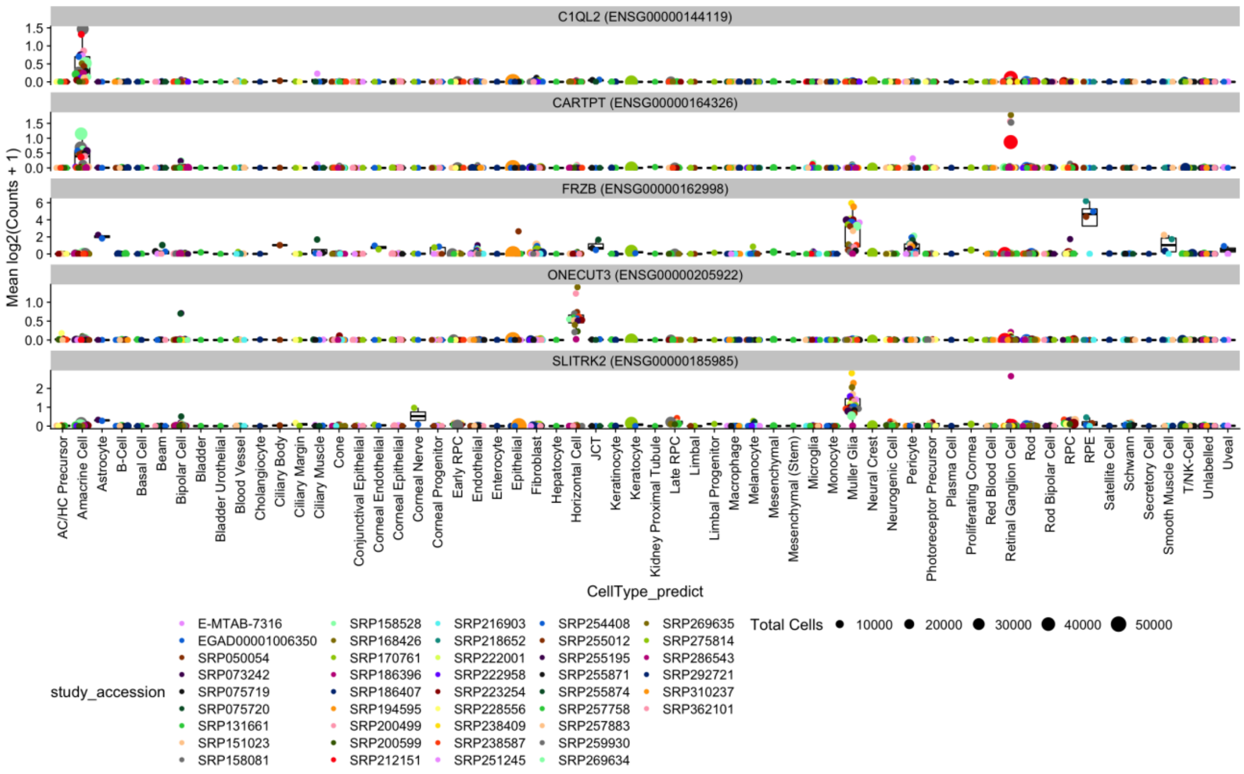

Supplemental Figure 8: Five hand-selected genes selected from Supplementary Figure 3 as having fairly unique expression to a celltype while having few pubmed citations. Plotted as expression/boxplots to confirm specificity at the study level.

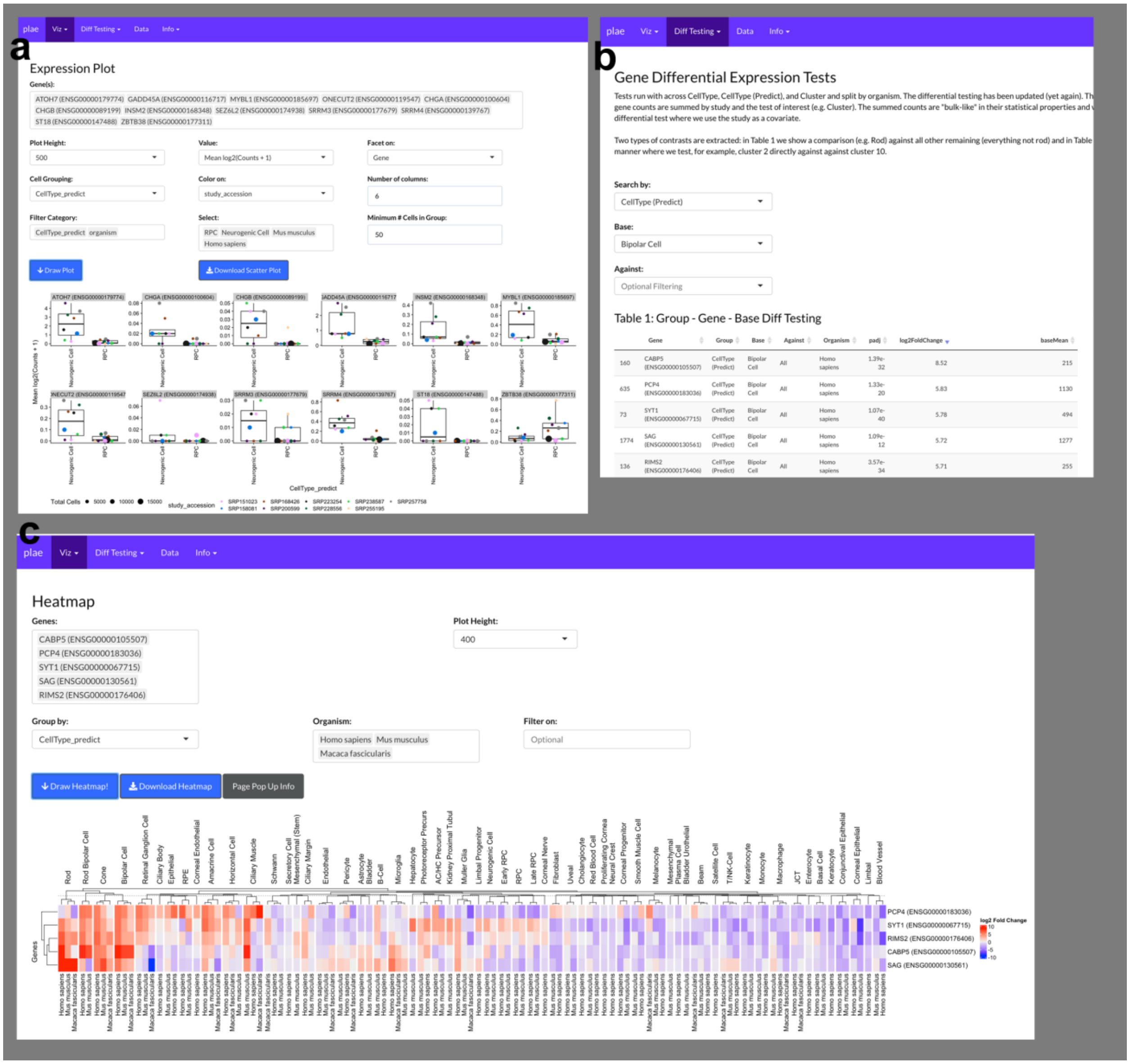

Supplemental Figure 9: a. Recreation of Figure 4 directly in the plae.nei.nih.gov web app. b. Identify potential bipolar cell markers with the precomputed differential testing and in c. visualize the top five genes with a Heatmap.

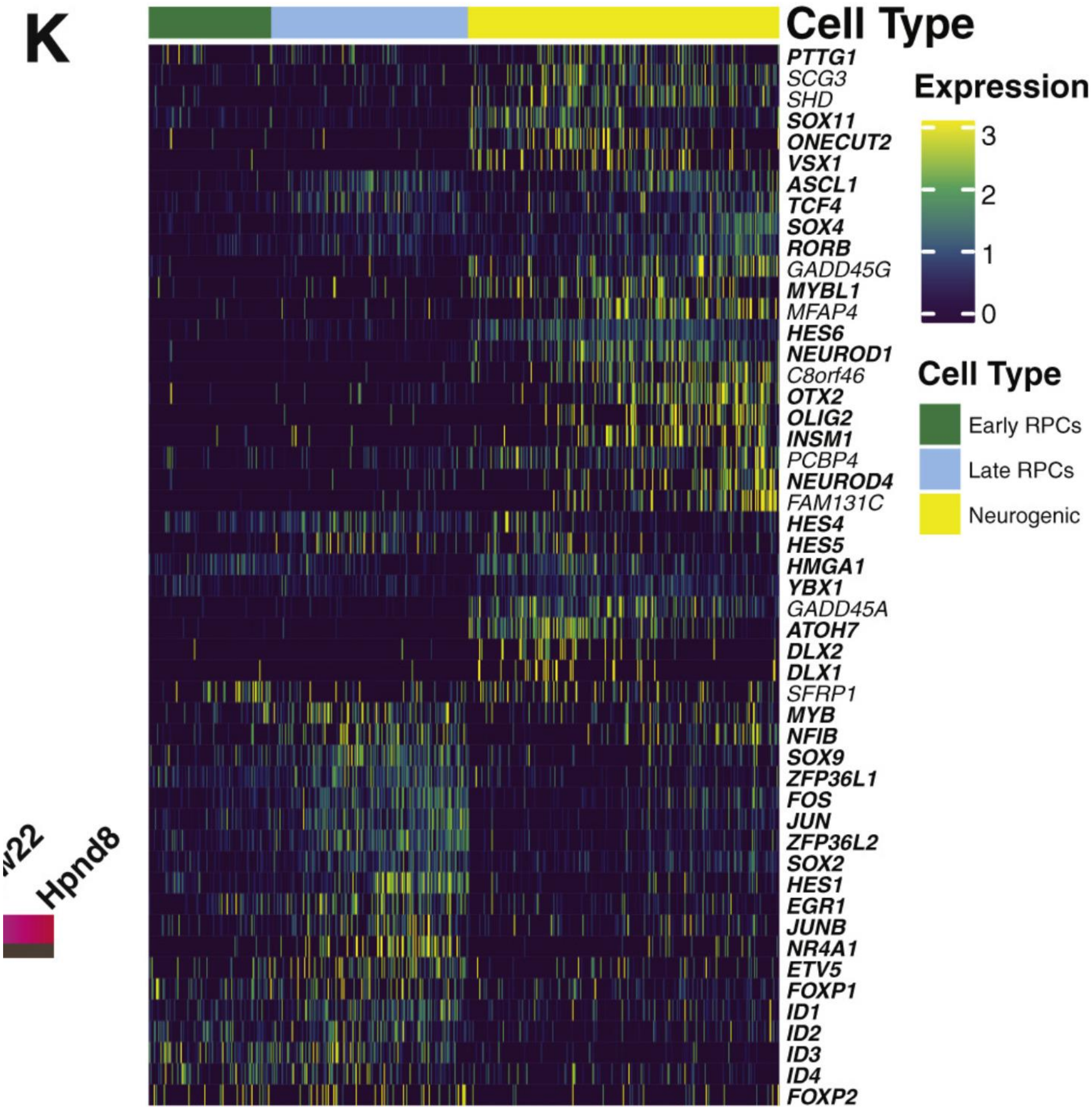

Supplemental Figure 10: Supplemental figure 2K from Lu et al showing their candidate RPC / Neurogenic differentially expressed genes

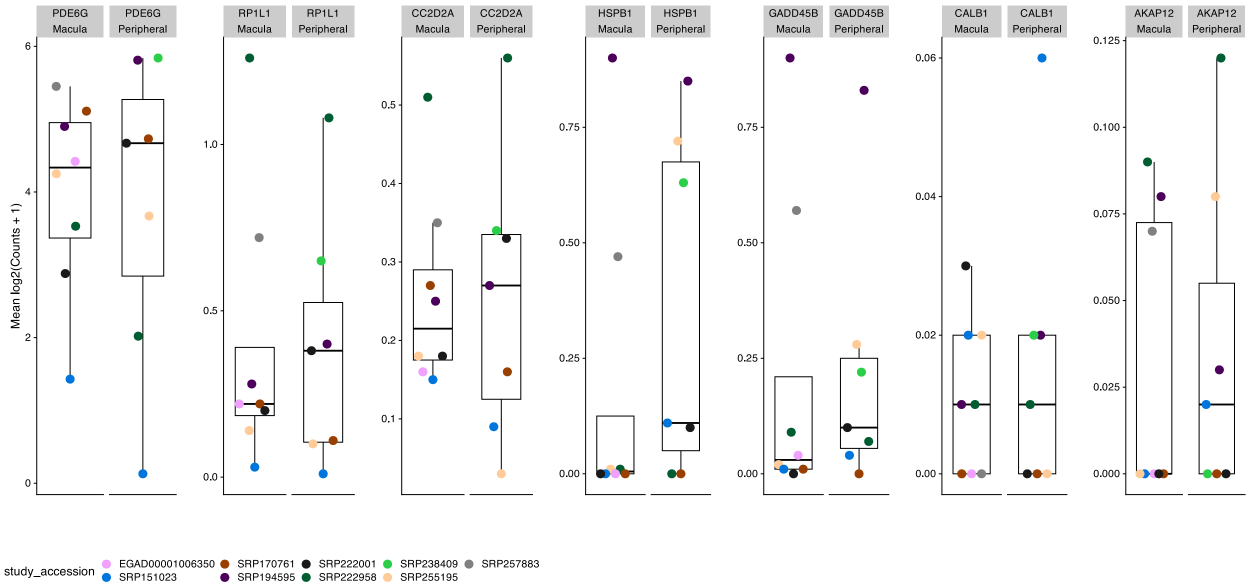

Supplemental Figure 11: Human cone fovea - peripheral differentially expressed genes are not differentially expressed in human rods.

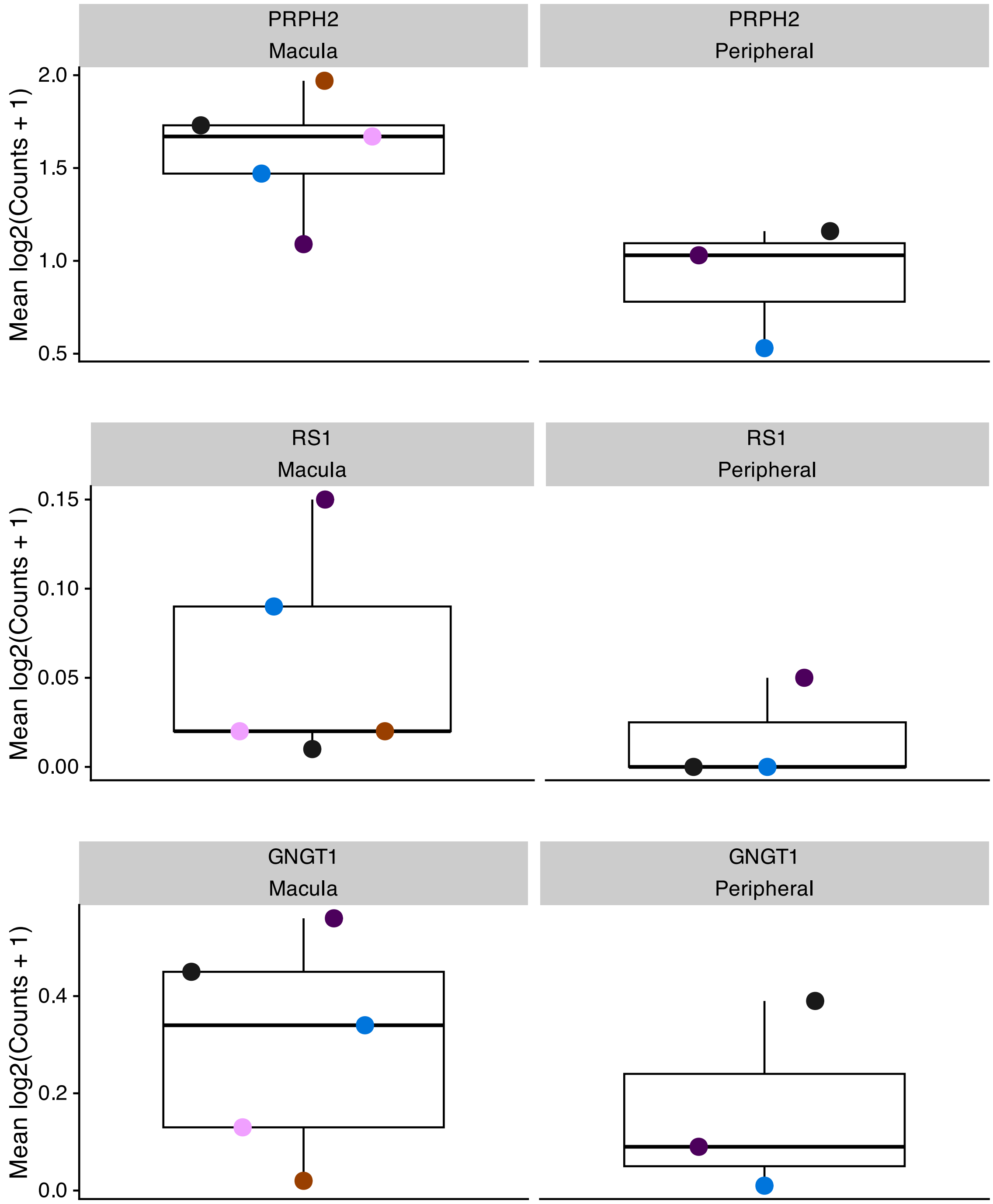

Supplemental Figure 12: Genes identified by Peng et al. and Yan et al. as macula / peripheral differentially expressed in cones.

organism	CellType	CellType_predict	Count	Ratio
Gallus gallus	Amacrine Cell	Amacrine Cell	6,483	0.98750952

organism	CellType	CellType_predict	Count	Ratio
Gallus gallus	Bipolar Cell	Bipolar Cell	10,681	0.99636194
Gallus gallus	Cone	Cone	1,112	0.98407080
Gallus gallus	Horizontal Cell	Amacrine Cell	30	0.01723148
Gallus gallus	Horizontal Cell	Horizontal Cell	1,694	0.97300402
Gallus gallus	Muller Glia	Muller Glia	3,033	0.99835418
Gallus gallus	Retinal Ganglion Cell	Retinal Ganglion Cell	6,880	0.99811403
Gallus gallus	Rod	Bipolar Cell	26	0.04227642
Gallus gallus	Rod	Cone	14	0.02276423
Gallus gallus	Rod	Rod	575	0.93495935
Homo sapiens	AC/HC Precursor	AC/HC Precursor	1,307	0.91462561
Homo sapiens	AC/HC Precursor	Amacrine Cell	49	0.03428971
Homo sapiens	AC/HC Precursor	Neurogenic Cell	35	0.02449265
Homo sapiens	AC/HC Precursor	Photoreceptor Precursor	15	0.01049685
Homo sapiens	Amacrine Cell	Amacrine Cell	7,776	0.98517674
Homo sapiens	Astrocyte	Astrocyte	287	0.95348837
Homo sapiens	Astrocyte	Muller Glia	14	0.04651163
Homo sapiens	B-Cell	B-Cell	7,970	0.98589807
Homo sapiens	Basal Cell	Basal Cell	736	0.99459459
Homo sapiens	Beam	Beam	3,052	0.99348958
Homo sapiens	Bipolar Cell	Bipolar Cell	3,790	0.96807152
Homo sapiens	Bipolar Cell	Muller Glia	48	0.01226054
Homo sapiens	Blood Vessel	Blood Vessel	3,773	0.91733528
Homo sapiens	Blood Vessel	Fibroblast	108	0.02625821
Homo sapiens	Blood Vessel	Keratocyte	44	0.01069779
Homo sapiens	Blood Vessel	Neural Crest	112	0.02723073
Homo sapiens	Cholangiocyte	Cholangiocyte	235	0.96311475
Homo sapiens	Cholangiocyte	Epithelial	5	0.02049180

organism	CellType	CellType_predict	Count	Ratio
Homo sapiens	Cholangiocyte	T/NK-Cell	3	0.01229508
Homo sapiens	Ciliary Body	Ciliary Body	1,378	0.93487110
Homo sapiens	Ciliary Body	Epithelial	23	0.01560380
Homo sapiens	Ciliary Body	Fibroblast	69	0.04681140
Homo sapiens	Ciliary Margin	B-Cell	4	0.01104972
Homo sapiens	Ciliary Margin	Ciliary Margin	324	0.89502762
Homo sapiens	Ciliary Margin	Corneal Epithelial	11	0.03038674
Homo sapiens	Ciliary Margin	Corneal Nerve	5	0.01381215
Homo sapiens	Ciliary Margin	Proliferating Cornea	9	0.02486188
Homo sapiens	Ciliary Muscle	Beam	37	0.22155689
Homo sapiens	Ciliary Muscle	Ciliary Muscle	88	0.52694611
Homo sapiens	Ciliary Muscle	Corneal Epithelial	3	0.01796407
Homo sapiens	Ciliary Muscle	Macrophage	2	0.01197605
Homo sapiens	Ciliary Muscle	Schwann	33	0.19760479
Homo sapiens	Cone	Cone	3,737	0.97343058
Homo sapiens	Conjunctival Epithelial	Conjunctival Epithelial	824	0.91150442
Homo sapiens	Conjunctival Epithelial	Corneal Epithelial	47	0.05199115
Homo sapiens	Corneal Endothelial	Corneal Endothelial	490	0.55872292
Homo sapiens	Corneal Endothelial	Corneal Epithelial	18	0.02052452
Homo sapiens	Corneal Endothelial	Corneal Nerve	33	0.03762828
Homo sapiens	Corneal Endothelial	Corneal Progenitor	56	0.06385405
Homo sapiens	Corneal Endothelial	Fibroblast	159	0.18129989
Homo sapiens	Corneal Endothelial	Keratocyte	25	0.02850627
Homo sapiens	Corneal Endothelial	Neural Crest	45	0.05131129
Homo sapiens	Corneal Endothelial	Proliferating Cornea	39	0.04446978
Homo sapiens	Corneal Epithelial	Corneal Epithelial	1,689	0.93057851
Homo sapiens	Corneal Epithelial	Fibroblast	27	0.01487603

organism	CellType	CellType_predict	Count	Ratio
Homo sapiens	Corneal Epithelial	Keratocyte	20	0.01101928
Homo sapiens	Corneal Epithelial	Neural Crest	33	0.01818182
Homo sapiens	Corneal Epithelial	Proliferating Cornea	19	0.01046832
Homo sapiens	Corneal Nerve	B-Cell	6	0.02714932
Homo sapiens	Corneal Nerve	Ciliary Margin	12	0.05429864
Homo sapiens	Corneal Nerve	Conjunctival Epithelial	6	0.02714932
Homo sapiens	Corneal Nerve	Corneal Endothelial	6	0.02714932
Homo sapiens	Corneal Nerve	Corneal Epithelial	11	0.04977376
Homo sapiens	Corneal Nerve	Corneal Nerve	122	0.55203620
Homo sapiens	Corneal Nerve	Fibroblast	13	0.05882353
Homo sapiens	Corneal Nerve	Keratocyte	8	0.03619910
Homo sapiens	Corneal Nerve	Melanocyte	9	0.04072398
Homo sapiens	Corneal Nerve	Neural Crest	14	0.06334842
Homo sapiens	Corneal Nerve	Proliferating Cornea	7	0.03167421
Homo sapiens	Corneal Progenitor	Corneal Progenitor	1,021	0.46409091
Homo sapiens	Corneal Progenitor	Fibroblast	782	0.35545455
Homo sapiens	Corneal Progenitor	Keratocyte	305	0.13863636
Homo sapiens	Corneal Progenitor	Neural Crest	23	0.01045455
Homo sapiens	Corneal Progenitor	Proliferating Cornea	30	0.01363636
Homo sapiens	Endothelial	Endothelial	7,653	0.98583022
Homo sapiens	Enterocyte	Enterocyte	1,720	0.99710145
Homo sapiens	Epithelial	B-Cell	27	0.02119309
Homo sapiens	Epithelial	Epithelial	1,157	0.90816327
Homo sapiens	Epithelial	T/NK-Cell	77	0.06043956
Homo sapiens	Fibroblast	Fibroblast	42,564	0.95340919
Homo sapiens	Fibroblast	Keratocyte	1,770	0.03964699
Homo sapiens	Horizontal Cell	Amacrine Cell	69	0.01354269

organism	CellType	CellType_predict	Count	Ratio
Homo sapiens	Horizontal Cell	Horizontal Cell	4,880	0.95780177
Homo sapiens	Horizontal Cell	RPC	58	0.01138371
Homo sapiens	JCT	Beam	14	0.02451839
Homo sapiens	JCT	JCT	549	0.96147110
Homo sapiens	JCT	Uveal	6	0.01050788
Homo sapiens	Keratinocyte	Keratinocyte	3,352	0.99672911
Homo sapiens	Keratocyte	Keratocyte	31,131	0.98316700
Homo sapiens	Limbal	Fibroblast	953	0.48523422
Homo sapiens	Limbal	Keratocyte	283	0.14409369
Homo sapiens	Limbal	Limbal	667	0.33961303
Homo sapiens	Limbal	Neural Crest	28	0.01425662
Homo sapiens	Limbal Progenitor	Conjunctival Epithelial	6	0.05172414
Homo sapiens	Limbal Progenitor	Corneal Epithelial	9	0.07758621
Homo sapiens	Limbal Progenitor	Corneal Progenitor	2	0.01724138
Homo sapiens	Limbal Progenitor	Fibroblast	10	0.08620690
Homo sapiens	Limbal Progenitor	Keratocyte	31	0.26724138
Homo sapiens	Limbal Progenitor	Limbal Progenitor	57	0.49137931
Homo sapiens	Macrophage	Endothelial	216	0.04655172
Homo sapiens	Macrophage	Macrophage	4,288	0.92413793
Homo sapiens	Melanocyte	Melanocyte	5,404	0.97827661
Homo sapiens	Microglia	Microglia	477	0.93164062
Homo sapiens	Microglia	T/NK-Cell	11	0.02148438
Homo sapiens	Monocyte	Macrophage	71	0.03338035
Homo sapiens	Monocyte	Monocyte	2,046	0.96191819
Homo sapiens	Muller Glia	Muller Glia	17,335	0.99746821
Homo sapiens	Neural Crest	Fibroblast	57	0.01477067
Homo sapiens	Neural Crest	Keratocyte	477	0.12360715

organism	CellType	CellType_predict	Count	Ratio
Homo sapiens	Neural Crest	Neural Crest	3,288	0.85203421
Homo sapiens	Neurogenic Cell	Neurogenic Cell	1,764	0.86217009
Homo sapiens	Neurogenic Cell	Photoreceptor Precursor	52	0.02541544
Homo sapiens	Neurogenic Cell	RPC	173	0.08455523
Homo sapiens	Pericyte	Fibroblast	41	0.01894640
Homo sapiens	Pericyte	Pericyte	2,084	0.96303142
Homo sapiens	Pericyte	Smooth Muscle Cell	25	0.01155268
Homo sapiens	Photoreceptor Precursor	Cone	34	0.01726765
Homo sapiens	Photoreceptor Precursor	Neurogenic Cell	53	0.02691722
Homo sapiens	Photoreceptor Precursor	Photoreceptor Precursor	1,750	0.88877603
Homo sapiens	Photoreceptor Precursor	Rod	98	0.04977146
Homo sapiens	Plasma Cell	Plasma Cell	1,582	0.99434318
Homo sapiens	Proliferating Cornea	Fibroblast	79	0.01932958
Homo sapiens	Proliferating Cornea	Keratocyte	332	0.08123318
Homo sapiens	Proliferating Cornea	Neural Crest	41	0.01003181
Homo sapiens	Proliferating Cornea	Proliferating Cornea	3,556	0.87007585
Homo sapiens	RPC	RPC	17,180	0.97663578
Homo sapiens	RPE	RPE	661	0.99698341
Homo sapiens	Red Blood Cell	Corneal Epithelial	22	0.03001364
Homo sapiens	Red Blood Cell	Fibroblast	21	0.02864939
Homo sapiens	Red Blood Cell	Keratocyte	17	0.02319236
Homo sapiens	Red Blood Cell	Neural Crest	11	0.01500682
Homo sapiens	Red Blood Cell	Proliferating Cornea	9	0.01227831
Homo sapiens	Red Blood Cell	RPC	22	0.03001364
Homo sapiens	Red Blood Cell	Red Blood Cell	523	0.71350614
Homo sapiens	Red Blood Cell	T/NK-Cell	84	0.11459754
Homo sapiens	Retinal Ganglion Cell	Amacrine Cell	86	0.01112549

organism	CellType	CellType_predict	Count	Ratio
Homo sapiens	Retinal Ganglion Cell	Retinal Ganglion Cell	7,598	0.98292367
Homo sapiens	Rod	Rod	40,144	0.99501797
Homo sapiens	Rod Bipolar Cell	Rod Bipolar Cell	210	0.99056604
Homo sapiens	Satellite Cell	Satellite Cell	2,293	0.99956408
Homo sapiens	Schwann	Beam	38	0.01230969
Homo sapiens	Schwann	Schwann	2,994	0.96987366
Homo sapiens	Secretory Cell	B-Cell	17	0.04829545
Homo sapiens	Secretory Cell	Epithelial	12	0.03409091
Homo sapiens	Secretory Cell	Fibroblast	6	0.01704545
Homo sapiens	Secretory Cell	Secretory Cell	297	0.84375000
Homo sapiens	Secretory Cell	Smooth Muscle Cell	4	0.01136364
Homo sapiens	Secretory Cell	T/NK-Cell	6	0.01704545
Homo sapiens	Smooth Muscle Cell	Fibroblast	63	0.01567554
Homo sapiens	Smooth Muscle Cell	Smooth Muscle Cell	3,879	0.96516546
Homo sapiens	T/NK-Cell	T/NK-Cell	23,825	0.99424112
Macaca fascicularis	Amacrine Cell	Amacrine Cell	18,926	0.99799620
Macaca fascicularis	Beam	Beam	1,766	0.99774011
Macaca fascicularis	Bipolar Cell	Bipolar Cell	6,029	0.99603502
Macaca fascicularis	Ciliary Muscle	Beam	16	0.01328904
Macaca fascicularis	Ciliary Muscle	Ciliary Muscle	1,170	0.97176080
Macaca fascicularis	Ciliary Muscle	Macrophage	14	0.01162791
Macaca fascicularis	Cone	Bipolar Cell	15	0.02846300
Macaca fascicularis	Cone	Cone	505	0.95825427
Macaca fascicularis	Cone	Rod	7	0.01328273
Macaca fascicularis	Endothelial	Beam	4	0.01133144
Macaca fascicularis	Endothelial	Endothelial	337	0.95467422
Macaca fascicularis	Endothelial	Pericyte	6	0.01699717

organism	CellType	CellType_predict	Count	Ratio
Macaca fascicularis	Horizontal Cell	Amacrine Cell	4	0.03278689
Macaca fascicularis	Horizontal Cell	Horizontal Cell	116	0.95081967
Macaca fascicularis	JCT	Beam	12	0.17142857
Macaca fascicularis	JCT	Fibroblast	3	0.04285714
Macaca fascicularis	JCT	JCT	54	0.77142857
Macaca fascicularis	JCT	Uveal	1	0.01428571
Macaca fascicularis	Macrophage	Macrophage	821	0.99878345
Macaca fascicularis	Melanocyte	Melanocyte	68	1.00000000
Macaca fascicularis	Microglia	Microglia	125	0.96899225
Macaca fascicularis	Microglia	Muller Glia	2	0.01550388
Macaca fascicularis	Muller Glia	Muller Glia	10,584	0.99380282
Macaca fascicularis	Pericyte	Pericyte	2,521	0.99723101
Macaca fascicularis	Retinal Ganglion Cell	Retinal Ganglion Cell	11,631	0.99939852
Macaca fascicularis	Rod	Amacrine Cell	20	0.01587302
Macaca fascicularis	Rod	Bipolar Cell	28	0.02222222
Macaca fascicularis	Rod	Muller Glia	13	0.01031746
Macaca fascicularis	Rod	Rod	1,185	0.94047619
Macaca fascicularis	Schwann	Beam	3	0.02238806
Macaca fascicularis	Schwann	Schwann	129	0.96268657
Macaca fascicularis	T/NK-Cell	Macrophage	39	0.31967213
Macaca fascicularis	T/NK-Cell	T/NK-Cell	82	0.67213115
Mus musculus	AC/HC Precursor	AC/HC Precursor	355	0.95687332
Mus musculus	AC/HC Precursor	Neurogenic Cell	6	0.01617251
Mus musculus	AC/HC Precursor	Retinal Ganglion Cell	6	0.01617251
Mus musculus	Amacrine Cell	Amacrine Cell	8,350	0.95943927
Mus musculus	Amacrine Cell	Bipolar Cell	92	0.01057107
Mus musculus	Amacrine Cell	Neurogenic Cell	181	0.02079743

organism	CellType	CellType_predict	Count	Ratio
Mus musculus	Astrocyte	Astrocyte	12	0.60000000
Mus musculus	Astrocyte	Muller Glia	7	0.35000000
Mus musculus	Astrocyte	Pericyte	1	0.05000000
Mus musculus	B-Cell	B-Cell	8,135	0.99926299
Mus musculus	Basal Cell	Basal Cell	4,391	0.99501473
Mus musculus	Beam	Beam	84	0.75675676
Mus musculus	Beam	Fibroblast	24	0.21621622
Mus musculus	Beam	Uveal	2	0.01801802
Mus musculus	Bipolar Cell	Bipolar Cell	12,847	0.98166119
Mus musculus	Bladder	Bladder	1,165	0.99148936
Mus musculus	Bladder Urothelial	Bladder Urothelial	1,145	0.99912740
Mus musculus	Ciliary Margin	Ciliary Margin	339	0.25260805
Mus musculus	Ciliary Margin	Early RPC	90	0.06706408
Mus musculus	Ciliary Margin	Neurogenic Cell	164	0.12220566
Mus musculus	Ciliary Margin	RPC	748	0.55737705
Mus musculus	Ciliary Muscle	Corneal Endothelial	1	0.03846154
Mus musculus	Ciliary Muscle	Epithelial	10	0.38461538
Mus musculus	Ciliary Muscle	Fibroblast	1	0.03846154
Mus musculus	Ciliary Muscle	Pericyte	9	0.34615385
Mus musculus	Ciliary Muscle	Uveal	5	0.19230769
Mus musculus	Cone	Bipolar Cell	53	0.02059852
Mus musculus	Cone	Cone	2,245	0.87252235
Mus musculus	Cone	Late RPC	100	0.03886514
Mus musculus	Cone	Neurogenic Cell	45	0.01748931
Mus musculus	Cone	Photoreceptor Precursor	32	0.01243684
Mus musculus	Cone	Rod	79	0.03070346
Mus musculus	Corneal Epithelial	Corneal Epithelial	223	0.75337838

organism	CellType	CellType_predict	Count	Ratio
Mus musculus	Corneal Epithelial	Epithelial	5	0.01689189
Mus musculus	Corneal Epithelial	Fibroblast	13	0.04391892
Mus musculus	Corneal Epithelial	Macrophage	3	0.01013514
Mus musculus	Corneal Epithelial	Melanocyte	7	0.02364865
Mus musculus	Corneal Epithelial	Uveal	41	0.13851351
Mus musculus	Early RPC	Early RPC	24,730	0.97689117
Mus musculus	Endothelial	Endothelial	3,117	0.99046711
Mus musculus	Epithelial	Epithelial	33,584	0.98541709
Mus musculus	Fibroblast	Epithelial	10	0.01683502
Mus musculus	Fibroblast	Fibroblast	561	0.94444444
Mus musculus	Fibroblast	Pericyte	10	0.01683502
Mus musculus	Hepatocyte	Hepatocyte	1,015	0.99901575
Mus musculus	Horizontal Cell	Amacrine Cell	146	0.11764706
Mus musculus	Horizontal Cell	Horizontal Cell	1,013	0.81627720
Mus musculus	Horizontal Cell	Neurogenic Cell	41	0.03303787
Mus musculus	Horizontal Cell	Retinal Ganglion Cell	24	0.01933924
Mus musculus	JCT	Fibroblast	4	0.02259887
Mus musculus	JCT	Uveal	173	0.97740113
Mus musculus	Keratinocyte	Basal Cell	35	0.01203576
Mus musculus	Keratinocyte	Keratinocyte	2,873	0.98796424
Mus musculus	Kidney Proximal Tubule	Hepatocyte	3	0.05454545
Mus musculus	Kidney Proximal Tubule	Kidney Proximal Tubule	52	0.94545455
Mus musculus	Late RPC	Early RPC	412	0.02351598
Mus musculus	Late RPC	Late RPC	16,726	0.95468037
Mus musculus	Late RPC	Neurogenic Cell	256	0.01461187
Mus musculus	Macrophage	Macrophage	1,629	0.97079857
Mus musculus	Macrophage	Monocyte	19	0.01132300

organism	CellType	CellType_predict	Count	Ratio
Mus musculus	Melanocyte	Melanocyte	538	0.99445471
Mus musculus	Mesenchymal	Mesenchymal	7,813	0.99948829
Mus musculus	Mesenchymal (Stem)	Mesenchymal (Stem)	1,082	0.98096102
Mus musculus	Microglia	Cone	1	0.07692308
Mus musculus	Microglia	Microglia	12	0.92307692
Mus musculus	Monocyte	Monocyte	509	0.99220273
Mus musculus	Muller Glia	Late RPC	601	0.16349293
Mus musculus	Muller Glia	Muller Glia	2,878	0.78291621
Mus musculus	Muller Glia	Neurogenic Cell	84	0.02285092
Mus musculus	Muller Glia	RPC	38	0.01033732
Mus musculus	Neurogenic Cell	Early RPC	183	0.02606466
Mus musculus	Neurogenic Cell	Late RPC	435	0.06195699
Mus musculus	Neurogenic Cell	Neurogenic Cell	6,102	0.86910696
Mus musculus	Neurogenic Cell	Photoreceptor Precursor	154	0.02193420
Mus musculus	Neurogenic Cell	RPC	87	0.01239140
Mus musculus	Pericyte	Endothelial	9	0.01080432
Mus musculus	Pericyte	Fibroblast	17	0.02040816
Mus musculus	Pericyte	Pericyte	797	0.95678271
Mus musculus	Photoreceptor Precursor	Neurogenic Cell	261	0.05683798
Mus musculus	Photoreceptor Precursor	Photoreceptor Precursor	4,079	0.88828397
Mus musculus	Photoreceptor Precursor	Retinal Ganglion Cell	59	0.01284843
Mus musculus	Photoreceptor Precursor	Rod	72	0.01567944
Mus musculus	RPC	Neurogenic Cell	33	0.01022622
Mus musculus	RPC	RPC	3,185	0.98698482
Mus musculus	Red Blood Cell	Red Blood Cell	1,742	0.98085586
Mus musculus	Retinal Ganglion Cell	Retinal Ganglion Cell	41,670	0.99770148
Mus musculus	Rod	Bipolar Cell	85	0.01017720

organism	CellType	CellType_predict	Count	Ratio
Mus musculus	Rod	Photoreceptor Precursor	117	0.01400862
Mus musculus	Rod	Rod	7,975	0.95486111
Mus musculus	Rod Bipolar Cell	Bipolar Cell	567	0.06406056
Mus musculus	Rod Bipolar Cell	Rod Bipolar Cell	8,284	0.93593944
Mus musculus	Schwann	Schwann	27	1.00000000
Mus musculus	Smooth Muscle Cell	Fibroblast	2	0.10000000
Mus musculus	Smooth Muscle Cell	Pericyte	17	0.85000000
Mus musculus	Smooth Muscle Cell	Smooth Muscle Cell	1	0.05000000
Mus musculus	T/NK-Cell	T/NK-Cell	5,373	0.99888455
Mus musculus	Uveal	Uveal	2,548	0.98951456

Supplemental Table 1: Count and ratio of cell type and predicted cell type labelling assignments split by organisms shows overall strong labelling performance. Cell type to predicted cell type comparisons with a ratio less than 0.01 were filtered out.

Citation	PMID	organism	study_accession	Count	Tissues
Balasubramanian R, Min X, Quinn P, Lo Giuduce Q, Tao C et al. Phase transition specified by a binary code patterns the vertebrate eye cup Sci Adv. 2021 Nov 12;7(46)	34757798	Mus musculus	SRP228556	8,463	Retina
Buenaventura DF, Corseri A, Emerson MM. Identification of Genes With Enriched Expression in Early Developing Mouse Cone Photoreceptors. Invest Ophthalmol Vis Sci 2019 Jul 1;60(8):2787-2799	31260032	Mus musculus	SRP200599	8,207	Retina
Clark BS, Stein-O'Brien GL, Shiau F, Cannon GH et al. Single-Cell RNA-Seq Analysis of Retinal Development Identifies NFI Factors as Regulating Mitotic Exit and Late-Born Cell Specification. Neuron 2019 Jun 19;102(6):1111-1126.e5	31128945	Mus musculus	SRP158081	127,939	Retina
Collin J, Queen R, Zerti D, Bojic S et al. A single cell atlas of human cornea that defines its development, limbal progenitor cells and their interactions with the immune cells. Ocul Surf 2021 Jul;21:279-298	33865984	Homo sapiens	SRP275814	100,472	Cornea

Citation	PMID	organism	study_accession	Count	Tissues
Cowan CS, Renner M, De Gennaro M, Gross-Sherf B et al. Cell Types of the Human Retina and Its Organoids at Single-Cell Resolution Cell Press 2020 Sep 17;182(6):1623-1640	32946783	Homo sapiens	EGAD00001006350	55,018	Retina, RPE-Choroid
Dani N, Herbst RH, McCabe C, Green GS et al. A cellular and spatial map of the choroid plexus across brain ventricles and ages. Cell 2021 May 27;184(11):3056-3074.e21	33932339	Mus musculus	SRP310237	64,671	Brain Choroid Plexus
Fadl BR, Brodie SA, Malasky M, Boland JF et al. An optimized protocol for retina single-cell RNA sequencing. Mol Vis 2020;26:705-717	33088174	Mus musculus	SRP269634	358	Retina
Fadl BR, Brodie SA, Malasky M, Boland JF et al. An optimized protocol for retina single-cell RNA sequencing. Mol Vis 2020;26:705-717	33088174	Mus musculus	SRP269635	8,516	Retina
Gautam P, Hamashima K, Chen Y, Zeng Y et al. Multi-species single-cell transcriptomic analysis of ocular compartment regulons. Nat Commun 2021 Sep 28;12(1):5675.	34584087	Homo sapiens	SRP255012	23,610	Cornea, Iris, RPE-Choroid, Retina, Sclera
He S, Wang LH, Liu Y, Li YQ et al. Single-cell transcriptome profiling of an adult human cell atlas of 15 major organs. Genome Biol 2020 Dec 7;21(1):294.	33287869	Homo sapiens	SRP292721	79,245	Trachea, Stomach, Spleen, Small.intestine, Skin, Rectum, Muscle, Marrow, Lymph.node, Liver, Heart, Esophagus, Common.bile.duct, Blood, Bladder
Heng JS, Hackett SF, Stein-O'Brien GL, Winer BL et al. Comprehensive analysis of a mouse model of spontaneous uveoretinitis using single-cell RNA sequencing. Proc Natl Acad Sci U S A 2019 Dec 16.	31843893	Mus musculus	SRP200499	15,461	Retina
Hu Y, Wang X, Hu B, Mao Y et al. Dissecting the transcriptome landscape of the human fetal neural retina and retinal pigment epithelium by single-cell RNA-seq analysis. PLoS Biol 2019 Jul;17(7):e3000365	31269016	Homo sapiens	SRP125998	8	Retina, RPE-Choroid
Lehmann GL, Hanke-Gogokhia C, Hu Y, Bareja R et al. Single-cell profiling reveals an endothelium-mediated immunomodulatory pathway in the eye choroid. J Exp Med 2020 Jun 1;217(6)	32196081	Mus musculus	SRP216903	9,607	RPE-Choroid
Ligocki A, Fury W, Gutierrez C, Adler, C, et al. Molecular characteristics and spatial distribution of adult human corneal cell subtypes Sci Rep. 2021 Aug 11;11(1):16323	34381080	Homo sapiens	SRP362101	12,289	Cornea
Lo Giudice Q, Leleu M, La Manno G, Fabre PJ. Single-cell transcriptional logic of cell-fate specification and axon guidance in early-born retinal neurons.	31399471	Mus musculus	SRP168426	5,040	Retina
Lo Giudice Q, Leleu M, La Manno G, Fabre PJ. Single-cell transcriptional logic of cell-fate specification and axon guidance in early-born retinal neurons. Development 2019 Sep 9;146(17)	31399471	Mus musculus	SRP186396	599	Retina
Lu Y, Shiau F, Yi W, Lu S et al. Single-Cell Analysis of Human Retina Identifies Evolutionarily Conserved and Species-Specific Mechanisms Controlling Development. Dev Cell 2020 May 18;53(4):473-491.e9	32386599	Homo sapiens	SRP151023	42,827	Retina
Lu Y, Shiau F, Yi W, Lu S et al. Single-Cell Analysis of Human Retina Identifies Evolutionarily Conserved and Species-Specific Mechanisms Controlling Development. Dev Cell 2020 May 18;53(4):473-491.e9	32386599	Homo sapiens	SRP223254	64,425	Retina

Citation	PMID	organism	study_accession	Count	Tissues
Lu Y, Shiau F, Yi W, Lu S et al. Single-Cell Analysis of Human Retina Identifies Evolutionarily Conserved and Species-Specific Mechanisms Controlling Development. Dev Cell 2020 May 18;53(4):473-491.e9.	32386599	Homo sapiens	SRP170761	5,002	Retina
Lukowski SW, Lo CY, Sharov AA et al. A single-cell transcriptome atlas of the adult human retina:e100811 (2019)	31436334	Homo sapiens	E-MTAB-7316	9,725	Retina
Macosko EZ, Basu A, Satija R, Nemesh J et al. Highly Parallel Genome-wide Expression Profiling of Individual Cells Using Nanoliter Droplets. Cell 2015 May 21;161(5):1202-1214	26000488	Mus musculus	SRP050054	12,092	Retina
Menon M, Mohammadi S, Davila-Velderrain J, Goods BA, Cadwell TD, Xing Y, Rachamimov AS, Shalek AK, Love JC, Kellis K, Hafler BP. Single-cell transcriptomic atlas of the human retina identifies cell types associated with age-related macular degeneration. Nature Communications 2019 Oct 25;10-4902	31653841	Homo sapiens	SRP222001	1,273	Retina
Menon M, Mohammadi S, Davila-Velderrain J, Goods BA, Cadwell TD, Xing Y, Rachamimov AS, Shalek AK, Love JC, Kellis K, Hafler BP. Single-cell transcriptomic atlas of the human retina identifies cell types associated with age-related macular degeneration. Nature Communications 2019 Oct 25;10-4902	31653841	Homo sapiens	SRP222958	3,894	Retina
O'Koren EG, Yu C, Klingeborn M, Wong AYW et al. Microglial Function Is Distinct in Different Anatomical Locations during Retinal Homeostasis and Degeneration. Immunity 2019 Mar 19;50(3):723-737.e7	30850344	Mus musculus	SRP186407	3,621	Retina
Patel G, Fury W, Yang H, et al. Molecular taxonomy of human ocular outflow tissues defined by single-cell transcriptomics Proc Natl Acad Sci U S A. 2020 Jun 9;117(23):12856-12867	32439707	Homo sapiens	SRP254408	10,618	Outflow Tract
Peng YR, Shekhar K, Yan W, Herrmann D et al. Molecular Classification and Comparative Taxonomics of Foveal and Peripheral Cells in Primate Retina. Cell2019 Feb 21;176(5):1222-1237.e22	30712875	Macaca fascicularis	SRP158528	85,327	Retina
Shekhar K, Lapan SW, Whitney IE, Tran NM et al. Comprehensive Classification of Retinal Bipolar Neurons by Single-Cell Transcriptomics. Cell 2016 Aug 25;166(5):1308-1323.e30	27565351	Mus musculus	SRP073242	246	Retina
Shekhar K, Lapan SW, Whitney IE, Tran NM et al. Comprehensive Classification of Retinal Bipolar Neurons by Single-Cell Transcriptomics. Cell 2016 Aug 25;166(5):1308-1323.e30	27565351	Mus musculus	SRP075719	24,158	Retina
Shekhar K, Lapan SW, Whitney IE, Tran NM et al. Comprehensive Classification of Retinal Bipolar Neurons by Single-Cell Transcriptomics. Cell 2016 Aug 25;166(5):1308-1323.e30	27565351	Mus musculus	SRP075720	337	Retina
Sridhar A, Hoshino A, Finkbeiner CR, Chitsazan A et al. Single-Cell Transcriptomic Comparison of Human Fetal Retina, hPSC-Derived Retinal Organoids, and Long-Term Retinal Cultures. Cell Rep 2020 Feb 4;30(5):1644-1659.e4	32023475	Homo sapiens	SRP238587	18,575	Retina
Swamy VS, Fufa TD, Hufnagel RB, McGaughey DM Building the Mega Single Cell Transcriptome Ocular Meta-Atlas Gigascience 2021 Oct 13;10(10)	34651173	Homo sapiens	SRP329495	1,544	RPE-Choroid

Citation	PMID	organism	study_accession	Count	Tissues
Tabula Muris Consortium., Overall coordination., Logistical coordination., Organ collection and processing. et al. Single-cell transcriptomics of 20 mouse organs creates a Tabula Muris. Nature 2018 Oct;562(7727):367-372	30283141	Mus musculus	SRP131661	60,587	Trachea, Lung, Limb_Muscle, Mammary_Gland, Thymus, Tongue, Bladder, Spleen, Kidney, Heart_and_Aorta, Marrow, Liver
Tran NM, Shekhar K, Whitney IE, Jacobi A et al. Single-Cell Profiles of Retinal Ganglion Cells Differing in Resilience to Injury Reveal Neuroprotective Genes. Neuron 2019 Dec 18;104(6):1039-1055.e12	31784286	Mus musculus	SRP212151	46,175	Retina
Voigt AP, Binkley E, Flamme-Wiese MJ, Zeng S et al. Single-Cell RNA Sequencing in Human Retinal Degeneration Reveals Distinct Glial Cell Populations. Cells 2020 Feb 13;9(2):438	32069977	Homo sapiens	SRP238409	1,195	Retina
Voigt AP, Mulfaul K, Mullin NK, Flamme-Wiese MJ et al. Single-cell transcriptomics of the human retinal pigment epithelium and choroid in health and macular degeneration. Proc Natl Acad Sci U S A 2019 Nov 26;116(48):24100-24107	31712411	Homo sapiens	SRP218652	12,634	RPE-Choroid
Voigt AP, Whitmore SS, Flamme-Wiese MJ, Riker MJ et al. Molecular characterization of foveal versus peripheral human retina by single-cell RNA sequencing. Exp Eye Res 2019 Jul;184:234-242	31075224	Homo sapiens	SRP194595	3,645	Retina
Voigt AP, Whitmore SS, Mulfaul K, Chirco KR et al. Bulk and single-cell gene expression analyses reveal aging human choriocapillaris has pro-inflammatory phenotype. Microvasc Res 2020 Sep;131:104031.	32531351	Homo sapiens	SRP257883	24,780	RPE-Choroid
Wu F, Bard JE, Kann J, Yergeau D et al. Single cell transcriptomics reveals lineage trajectory of retinal ganglion cells in wild-type and Atoh7-null retinas. Nat Commun 2021 Mar 5;12(1):1465	33674582	Mus musculus	SRP257758	42,692	Retina
Yamagata M, Yan W, Sanes JR. A cell atlas of the chick retina based on single-cell transcriptomics. Elife 2021 Jan 4;10	33393903	Gallus gallus	SRP286543	37,498	Retina
Yan W, Laboulaye MA, Tran NM, Whitney IE et al. Mouse Retinal Cell Atlas: Molecular Identification of over Sixty Amacrine Cell Types. J Neurosci 2020 Jul 1;40(27):5177-5195	32457074	Mus musculus	SRP259930	44,560	Retina
Yan W, Peng YR, van Zyl T, Regev A et al. Cell Atlas of The Human Fovea and Peripheral Retina. Sci Rep 2020 Jun 17;10(1):9802	32555229	Homo sapiens	SRP255195	28,924	Retina
van Zyl T, Yan W, McAdams A, Peng YR et al. Cell atlas of aqueous humor outflow pathways in eyes of humans and four model species provides insight into glaucoma pathogenesis. Proc Natl Acad Sci U S A 2020 May 12;117(19):10339-10349	32341164	Homo sapiens	SRP255871	10,858	Outflow Tract
van Zyl T, Yan W, McAdams A, Peng YR et al. Cell atlas of aqueous humor outflow pathways in eyes of humans and four model species provides insight into glaucoma pathogenesis. Proc Natl Acad Sci U S A 2020 May 12;117(19):10339-10349	32341164	Mus musculus	SRP251245	14,827	Outflow Tract
van Zyl T, Yan W, McAdams A, Peng YR et al. Cell atlas of aqueous humor outflow pathways in eyes of humans and four model species provides insight into glaucoma pathogenesis. Proc Natl Acad Sci U S A 2020 May 12;117(19):10339-10349	32341164	Macaca fascicularis	SRP255874	4,499	Outflow Tract

Supplemental Table 2: Studies, tissues, and number of cells across scEiaD

CellType (Predict)	Homo sapiens	Mus musculus	Macaca fascicularis
AC/HC Precursor	134	106	0
Amacrine Cell	1,238	1,169	27
Astrocyte	134	0	0
B-Cell	718	48	0
Beam	43	2	72
Bipolar Cell	1,101	932	18
Blood Vessel	479	35	0
Ciliary Body	16	0	0
Ciliary Margin	62	0	0
Ciliary Muscle	48	94	95
Cone	949	661	75
Conjunctival Epithelial	197	27	0
Corneal Epithelial	285	15	0
Corneal Nerve	51	0	0
Corneal Progenitor	28	9	0
Early RPC	123	260	0
Endothelial	1,028	1,215	393
Epithelial	204	49	0
Fibroblast	867	209	0
Horizontal Cell	685	726	23
JCT	5	0	83
Keratocyte	3	0	0
Late RPC	145	315	0
Limbal	115	0	0
Macrophage	1,109	596	166
Melanocyte	453	97	63
Microglia	1,081	758	234

CellType (Predict)	Homo sapiens	Mus musculus	Macaca fascicularis
Monocyte	1,026	38	0
Muller Glia	1,060	927	95
Neurogenic Cell	71	340	0
Pericyte	565	752	337
Photoreceptor Precursor	43	75	0
Proliferating Cornea	4	0	0
Red Blood Cell	280	260	0
Retinal Ganglion Cell	1,244	1,285	56
Rod	995	612	64
Rod Bipolar Cell	414	154	0
RPC	151	463	0
RPE	628	10	0
Schwann	629	36	85
Smooth Muscle Cell	340	40	0
T/NK-Cell	837	43	95
Corneal Endothelial	0	0	0
Limbal Progenitor	0	0	0
Neural Crest	0	0	0
Uveal	0	0	0

Supplemental Table 3: Counts of significantly differentially expressed genes for each cell type across the organisms

CellType (Predict)	Gallus gallus	Homo sapiens	Macaca fascicularis	Mus musculus	Well Supported
AC/HC Precursor	0	3	0	3	Yes
Amacrine Cell	1	8	1	11	Yes
Astrocyte	0	2	0	0	No
B-Cell	0	5	0	0	No
Beam	0	3	1	1	No

CellType (Predict)	Gallus gallus	Homo sapiens	Macaca fascicularis	Mus musculus	Well Supported
Bipolar Cell	1	11	1	12	Yes
Blood Vessel	0	3	0	1	No
Ciliary Body	0	1	0	0	No
Ciliary Margin	0	1	0	1	No
Ciliary Muscle	0	2	1	1	No
Cone	1	8	1	9	Yes
Conjunctival Epithelial	0	3	0	1	No
Corneal Endothelial	0	2	0	0	No
Corneal Epithelial	0	4	0	1	No
Corneal Nerve	0	2	0	0	No
Corneal Progenitor	0	4	0	1	No
Early RPC	0	4	0	5	Yes
Endothelial	0	10	2	7	Yes
Epithelial	0	3	0	1	No
Fibroblast	0	10	0	3	Yes
Horizontal Cell	1	5	1	8	Yes
JCT	0	2	1	0	No
Keratocyte	0	2	0	0	No
Late RPC	0	3	0	4	Yes
Limbal	0	2	0	0	No
Limbal Progenitor	0	1	0	0	No
Macrophage	0	5	1	3	Yes
Melanocyte	0	8	1	2	Yes
Microglia	0	11	1	5	Yes
Monocyte	0	4	0	0	No
Muller Glia	1	12	1	6	Yes
Neural Crest	0	1	0	0	No
Neurogenic Cell	0	3	0	5	Yes
Pericyte	0	7	2	5	Yes
Photoreceptor Precursor	0	2	0	3	Yes

CellType (Predict)	Gallus gallus	Homo sapiens	Macaca fascicularis	Mus musculus	Well Supported
Proliferating Cornea	0	1	0	0	No
Red Blood Cell	0	2	0	1	No
Retinal Ganglion Cell	1	8	1	11	Yes
Rod	1	11	1	9	Yes
Rod Bipolar Cell	0	3	0	2	Yes
RPC	0	4	0	5	Yes
RPE	0	4	0	1	No
Schwann	0	5	1	1	No
Smooth Muscle Cell	0	2	0	1	No
T/NK-Cell	0	7	1	0	No
Uveal	0	1	0	1	No

Supplemental Table 4: Counts of independent studies for each cell type across the organisms

Gene	baseMean	log2FoldChange	lfcSE	stat	pvalue	padj	Against	Group	Base	Organism	Gene Name
GPX3 (ENSG00000211445)	1,543.579242	-7.8634099	1.1863587	-6.628189	0.00000000003398297	0.000004292049	RPC	CellType (Predict)	Neurogenic Cell	Homo sapiens	glutathione peroxidase 3
GDAP1L1 (ENSG00000124194)	96.212726	4.9984093	0.7737079	6.460331	0.00000000010447410	0.0000014505184	RPC	CellType (Predict)	Neurogenic Cell	Mus musculus	ganglioside induced differentiation associated protein 1 like 1
ANKS1A (ENSG00000064999)	70.520457	3.2606928	0.5253277	6.206969	0.00000000054016102	0.0000037497978	RPC	CellType (Predict)	Neurogenic Cell	Mus musculus	ankyrin repeat and sterile alpha motif domain containing 1A
SRRM4 (ENSG00000139767)	205.964623	3.4039678	0.5795381	5.873588	0.00000000426463363	0.0000197367244	RPC	CellType (Predict)	Neurogenic Cell	Mus musculus	serine/arginine repetitive matrix 4
IFI16 (ENSG00000163565)	323.574830	-4.2790163	0.7300608	-5.861178	0.00000000459594801	0.0000290234117	RPC	CellType (Predict)	Neurogenic Cell	Homo sapiens	interferon gamma inducible protein 16
AOC2 (ENSG00000131480)	11.972124	-7.1836169	1.2428473	-5.779967	0.00000000747150637	0.0000291444078	RPC	CellType (Predict)	Neurogenic Cell	Homo sapiens	amine oxidase copper containing 2
CRYAB (ENSG00000109846)	3,547.895029	-6.0958525	1.0612656	-5.743946	0.00000000924951861	0.0000291444078	RPC	CellType (Predict)	Neurogenic Cell	Homo sapiens	crystallin alpha B
GSN (ENSG00000148180)	1,499.714724	-5.3396975	0.9357352	-5.706419	0.00000001153777029	0.0000291444078	RPC	CellType (Predict)	Neurogenic Cell	Homo sapiens	gelsolin
KLHL35 (ENSG00000149243)	18.847041	4.6798418	0.8440797	5.544313	0.00000002951111950	0.0000621209065	RPC	CellType (Predict)	Neurogenic Cell	Homo sapiens	kelch like family member 35
SERPINE1 (ENSG00000106366)	98.631353	-7.0564314	1.3087530	-5.391721	0.00000006978590340	0.0001259137086	RPC	CellType (Predict)	Neurogenic Cell	Homo sapiens	serpin family E member 1
AIG1 (ENSG00000146416)	205.234584	-1.8688703	0.3487790	-5.358322	0.00000008399837293	0.0002063093259	RPC	CellType (Predict)	Neurogenic Cell	Mus musculus	androgen induced 1
STXBP1 (ENSG00000136854)	457.770439	2.6000846	0.4848393	5.362776	0.00000008195263853	0.0002063093259	RPC	CellType (Predict)	Neurogenic Cell	Mus musculus	syntaxin binding protein 1
SCG3 (ENSG00000104112)	331.050757	4.2925292	0.8045932	5.335030	0.00000009552851636	0.0002063093259	RPC	CellType (Predict)	Neurogenic Cell	Mus musculus	secretogranin III
GADD45A (ENSG00000116717)	436.064864	3.4618691	0.6507807	5.319563	0.00000010401651408	0.0002063093259	RPC	CellType (Predict)	Neurogenic Cell	Mus musculus	growth arrest and DNA damage inducible alpha
HES6 (ENSG00000144485)	715.102105	2.2577388	0.4281041	5.273807	0.00000013362226717	0.0002319014447	RPC	CellType (Predict)	Neurogenic Cell	Mus musculus	hes family bHLH transcription factor 6
TCIM (ENSG00000176907)	208.253555	-7.1537012	1.3730531	-5.210069	0.00000018877043430	0.0002980213231	RPC	CellType (Predict)	Neurogenic Cell	Homo sapiens	transcriptional and immune response regulator
CHGB (ENSG00000089199)	987.626445	4.7736436	0.9282670	5.142533	0.00000027105905905	0.0004181537751	RPC	CellType (Predict)	Neurogenic Cell	Mus musculus	chromogranin B
BCL9L (ENSG00000186174)	68.770819	-1.8429966	0.3599105	-5.120708	0.00000030439008959	0.0004226152004	RPC	CellType (Predict)	Neurogenic Cell	Mus musculus	BCL9 like
EMP1 (ENSG00000134531)	490.383491	-6.3546118	1.2454585	-5.102227	0.00000033567972644	0.0004239634945	RPC	CellType (Predict)	Neurogenic Cell	Homo sapiens	epithelial membrane protein 1
PCBP4 (ENSG00000090097)	441.078660	3.1538110	0.6176134	5.106448	0.00000032827061024	0.0004239634945	RPC	CellType (Predict)	Neurogenic Cell	Homo sapiens	poly(rC) binding protein 4
SERPINB6 (ENSG00000124570)	234.810919	-1.7487510	0.3515971	-4.973736	0.00000065674806284	0.0006382466210	RPC	CellType (Predict)	Neurogenic Cell	Homo sapiens	serpin family B member 6
GPRC5A (ENSG00000013588)	155.047295	-5.3365968	1.0729679	-4.973678	0.00000065694426551	0.0006382466210	RPC	CellType (Predict)	Neurogenic Cell	Homo sapiens	G protein-coupled receptor class C group 5 member A
GRK5 (ENSG00000198873)	10.111810	3.4265832	0.6908065	4.960265	0.00000070397090072	0.0007518409220	RPC	CellType (Predict)	Neurogenic Cell	Mus musculus	G protein-coupled receptor kinase 5
PLEK (ENSG00000115956)	153.752137	-4.0364385	0.8130899	-4.964320	0.00000068942197462	0.0007518409220	RPC	CellType (Predict)	Neurogenic Cell	Mus musculus	pleckstrin
TNFRSF25 (ENSG00000215788)	7.703349	-3.5558162	0.7152974	-4.971102	0.00000066573553795	0.0007518409220	RPC	CellType (Predict)	Neurogenic Cell	Mus musculus	TNF receptor superfamily member 25

Gene	baseMean	log2FoldChange	lfcSE	stat	pvalue	padj	Against	Group	Base	Organism	Gene Name
MYT1 (ENSG00000196132)	44.185177	4.5537335	0.9262155	4.916495	0.00000088107523021	0.0008155232331	RPC	CellType (Predict)	Neurogenic Cell	Mus musculus	myelin transcription factor 1
BTG2 (ENSG00000159388)	2,720.906214	2.6477991	0.5378421	4.923004	0.00000085225576222	0.0008155232331	RPC	CellType (Predict)	Neurogenic Cell	Mus musculus	BTG anti-proliferation factor 2
CHGA (ENSG00000100604)	44.645711	5.0309733	1.0258146	4.904369	0.00000093728125657	0.0008455615907	RPC	CellType (Predict)	Neurogenic Cell	Homo sapiens	chromogranin A
SPARCL1 (ENSG00000152583)	1,155.226527	-6.6972516	1.3703503	-4.887255	0.00000102251570413	0.0008609582229	RPC	CellType (Predict)	Neurogenic Cell	Homo sapiens	SPARC like 1
PILRA (ENSG00000085514)	25.756732	5.0411466	1.0386350	4.853627	0.00000121223888922	0.0010519202961	RPC	CellType (Predict)	Neurogenic Cell	Mus musculus	paired immunoglobin like type 2 receptor alpha
CEBPD (ENSG00000221869)	1,749.339800	-4.3402632	0.9056345	-4.792511	0.00000164706884585	0.0013001549702	RPC	CellType (Predict)	Neurogenic Cell	Homo sapiens	CCAAT enhancer binding protein delta
INSM2 (ENSG00000168348)	44.455221	3.9033233	0.8254155	4.728920	0.00000225717715040	0.0018434498562	RPC	CellType (Predict)	Neurogenic Cell	Mus musculus	INSM transcriptional repressor 2
PMM1 (ENSG00000100417)	379.302793	-1.8521459	0.3946545	-4.693082	0.00000269119837049	0.0020758110098	RPC	CellType (Predict)	Neurogenic Cell	Mus musculus	phosphomannomutase 1
RXYLT1 (ENSG00000118600)	173.769263	-1.0295474	0.2200599	-4.678487	0.00000288999591861	0.0021118264913	RPC	CellType (Predict)	Neurogenic Cell	Mus musculus	ribitol xylosyltransferase 1
CHGA (ENSG00000100604)	255.241221	3.5807162	0.7690454	4.656053	0.00000322329107499	0.0021310558707	RPC	CellType (Predict)	Neurogenic Cell	Mus musculus	chromogranin A
ANKRD33B (ENSG00000164236)	94.428956	4.2610185	0.9264188	4.599452	0.00000423604003016	0.0026733263536	RPC	CellType (Predict)	Neurogenic Cell	Mus musculus	ankyrin repeat domain 33B
SOGA3 (ENSG00000214338)	152.478683	2.0619477	0.4502024	4.580046	0.00000464873191964	0.0026892914155	RPC	CellType (Predict)	Neurogenic Cell	Mus musculus	SOGA family member 3
GRHL3 (ENSG00000158055)	20.507443	4.7998241	1.0464785	4.586644	0.00000450428356185	0.0026892914155	RPC	CellType (Predict)	Neurogenic Cell	Mus musculus	grainyhead like transcription factor 3
ONECUT2 (ENSG00000119547)	91.917237	5.0799142	1.0968651	4.631303	0.00000363372072212	0.0026996407483	RPC	CellType (Predict)	Neurogenic Cell	Homo sapiens	one cut homeobox 2
IGFBP3 (ENSG00000146674)	162.874737	-5.6083391	1.2298900	-4.560033	0.00000511455864809	0.0035514962958	RPC	CellType (Predict)	Neurogenic Cell	Homo sapiens	insulin like growth factor binding protein 3
SPHK1 (ENSG00000176170)	80.964013	-4.1703168	0.9163799	-4.550860	0.00000534271018378	0.0035514962958	RPC	CellType (Predict)	Neurogenic Cell	Homo sapiens	sphingosine kinase 1
CSRNP1 (ENSG00000144655)	250.347172	-3.4058561	0.7515300	-4.531896	0.00000584565283947	0.0036915297681	RPC	CellType (Predict)	Neurogenic Cell	Homo sapiens	cysteine and serine rich nuclear protein 1
CRX (ENSG00000105392)	400.843584	4.1897375	0.9328786	4.491192	0.00000708255132805	0.0036921722787	RPC	CellType (Predict)	Neurogenic Cell	Mus musculus	cone-rod homeobox
LRP11 (ENSG00000120256)	114.362456	2.7036561	0.6005283	4.502130	0.00000672759252516	0.0036921722787	RPC	CellType (Predict)	Neurogenic Cell	Mus musculus	LDL receptor related protein 11
GADD45G (ENSG00000130222)	747.860185	2.8614795	0.6427064	4.452235	0.00000849812768709	0.0039586361741	RPC	CellType (Predict)	Neurogenic Cell	Mus musculus	growth arrest and DNA damage inducible gamma
SLC37A4 (ENSG00000137700)	70.156679	-1.3104561	0.2944292	-4.450836	0.00000855366502607	0.0039586361741	RPC	CellType (Predict)	Neurogenic Cell	Mus musculus	solute carrier family 37 member 4
SHF (ENSG00000138606)	49.731352	2.4726314	0.5573668	4.436273	0.00000915295848001	0.0040993443721	RPC	CellType (Predict)	Neurogenic Cell	Mus musculus	Src homology 2 domain containing F
TRIM9 (ENSG00000100505)	58.106325	3.2013787	0.7229587	4.428163	0.00000950391772470	0.0041235123028	RPC	CellType (Predict)	Neurogenic Cell	Mus musculus	tripartite motif containing 9
NDST2 (ENSG00000272916)	30.763896	-1.1686684	0.2656311	-4.399592	0.00001084545747197	0.0045629797437	RPC	CellType (Predict)	Neurogenic Cell	Mus musculus	N-deacetylase and N-sulfotransferase 2
SRRM3 (ENSG00000177679)	134.162569	3.4893988	0.7948832	4.389826	0.00001134413238884	0.0046324098261	RPC	CellType (Predict)	Neurogenic Cell	Mus musculus	serine/arginine repetitive matrix 3
FLT1 (ENSG00000102755)	306.585477	-4.9796609	1.1459777	-4.345338	0.00001390611800621	0.0053447384519	RPC	CellType (Predict)	Neurogenic Cell	Mus musculus	fms related receptor tyrosine kinase 1
IGFBP6 (ENSG00000167779)	830.712689	-4.7133998	1.0611410	-4.441822	0.00000892002363700	0.0053647570731	RPC	CellType (Predict)	Neurogenic Cell	Homo sapiens	insulin like growth factor binding protein 6
ZBTB18 (ENSG00000179456)	89.654314	2.0442196	0.4739395	4.313250	0.00001608720769496	0.0058241768751	RPC	CellType (Predict)	Neurogenic Cell	Mus musculus	zinc finger and BTB domain containing 18
ME1 (ENSG00000065833)	66.870750	-3.9214464	0.8884530	-4.413792	0.00001015757330253	0.0058313704914	RPC	CellType (Predict)	Neurogenic Cell	Homo sapiens	malic enzyme 1
CHPF (ENSG00000123989)	122.886290	-2.6342927	0.5984122	-4.402137	0.00001071896261228	0.0058861085997	RPC	CellType (Predict)	Neurogenic Cell	Homo sapiens	chondroitin polymerizing factor
HELZ2 (ENSG00000130589)	15.351333	-5.4745691	1.2759702	-4.290515	0.00001782594566511	0.0058927483242	RPC	CellType (Predict)	Neurogenic Cell	Mus musculus	helicase with zinc finger 2
MYO1D (ENSG00000176658)	32.230821	3.4772694	0.8085009	4.300885	0.00001701174930842	0.0058927483242	RPC	CellType (Predict)	Neurogenic Cell	Mus musculus	myosin ID
YPEL2 (ENSG00000175155)	75.893847	2.5707234	0.5868596	4.380475	0.00001184210907063	0.0061212964182	RPC	CellType (Predict)	Neurogenic Cell	Homo sapiens	yippee like 2
IFI44L (ENSG00000137959)	76.237136	-5.5873362	1.2769654	-4.375480	0.00001211658040014	0.0061212964182	RPC	CellType (Predict)	Neurogenic Cell	Homo sapiens	interferon induced protein 44 like
GADD45A (ENSG00000116717)	400.777542	2.5260166	0.5814156	4.344597	0.00001395313867247	0.0067780054397	RPC	CellType (Predict)	Neurogenic Cell	Homo sapiens	growth arrest and DNA damage inducible alpha
IGFBP7 (ENSG00000163453)	2,236.313934	-4.7692655	1.1082039	-4.303599	0.00001680455439845	0.0071470227933	RPC	CellType (Predict)	Neurogenic Cell	Homo sapiens	insulin like growth factor binding protein 7
MYC (ENSG00000136997)	207.043729	-4.1462357	0.9641771	-4.300284	0.00001705790030821	0.0071470227933	RPC	CellType (Predict)	Neurogenic Cell	Homo sapiens	MYC proto-oncogene, bHLH transcription factor
GADD45B (ENSG00000099860)	2,366.919075	-3.6594368	0.8489452	-4.310569	0.00001628349886260	0.0071470227933	RPC	CellType (Predict)	Neurogenic Cell	Homo sapiens	growth arrest and DNA damage inducible beta
NHLH1 (ENSG00000171786)	18.291318	4.6315532	1.0753664	4.306954	0.00001655182190704	0.0071470227933	RPC	CellType (Predict)	Neurogenic Cell	Homo sapiens	nescient helix-loop-helix 1
ZNF331 (ENSG00000130844)	274.988445	-3.0725719	0.7155373	-4.294077	0.00001754217787748	0.0071470227933	RPC	CellType (Predict)	Neurogenic Cell	Homo sapiens	zinc finger protein 331
TTC30B (ENSG00000196659)	29.682515	-1.6789354	0.3978933	-4.219562	0.00002447776352481	0.0079034713669	RPC	CellType (Predict)	Neurogenic Cell	Homo sapiens	tetratricopeptide repeat domain 30B
PRDM1 (ENSG00000057657)	143.314724	3.8188439	0.9074056	4.208530	0.00002570373568557	0.0079304592502	RPC	CellType (Predict)	Neurogenic Cell	Mus musculus	PR/SET domain 1
CELF3 (ENSG00000159409)	355.991577	3.3173986	0.7878978	4.210443	0.00002548705160780	0.0079304592502	RPC	CellType (Predict)	Neurogenic Cell	Mus musculus	CUGBP Elav-like family member 3
NEK6 (ENSG00000119408)	42.293283	-2.6831035	0.6316641	-4.247674	0.00002160010623883	0.0085252919311	RPC	CellType (Predict)	Neurogenic Cell	Homo sapiens	NIMA related kinase 6
CAMKK1 (ENSG00000004660)	40.688541	3.0787115	0.7361866	4.181971	0.00002889923457462	0.0085815492694	RPC	CellType (Predict)	Neurogenic Cell	Mus musculus	calcium/calmodulin dependent protein kinase kinase 1
HTRA1 (ENSG00000166033)	18.793325	5.2185837	1.2482300	4.180787	0.00002905018839409	0.0085815492694	RPC	CellType (Predict)	Neurogenic Cell	Mus musculus	HtrA serine peptidase 1
RGCC (ENSG00000102760)	622.417782	-4.7501096	1.1223631	-4.232240	0.00002313756855521	0.0088553785107	RPC	CellType (Predict)	Neurogenic Cell	Homo sapiens	regulator of cell cycle

Gene	baseMean	log2FoldChange	lfcSE	stat	pvalue	padj	Against	Group	Base	Organism	Gene Name
PTGDS (ENSG00000107317)	3,340.130519	-5.8720853	1.3930413	-4.215299	0.00002494476327631	0.0090014960051	RPC	CellType (Predict)	Neurogenic Cell	Homo sapiens	prostaglandin D2 synthase
ZBTB38 (ENSG00000177311)	216.704372	-2.1134652	0.5012369	-4.216499	0.00002481240759094	0.0090014960051	RPC	CellType (Predict)	Neurogenic Cell	Homo sapiens	zinc finger and BTB domain containing 38
GPRIN3 (ENSG00000185477)	58.544286	3.8857757	0.9282008	4.186353	0.00002834728895257	0.0092358506248	RPC	CellType (Predict)	Neurogenic Cell	Homo sapiens	GPRIN family member 3
SRRM3 (ENSG00000177679)	57.377466	3.2513693	0.7757799	4.191097	0.00002776083640121	0.0092358506248	RPC	CellType (Predict)	Neurogenic Cell	Homo sapiens	serine/arginine repetitive matrix 3
ATOH7 (ENSG00000179774)	104.981119	4.9838271	1.1866980	4.199743	0.00002672176902843	0.0092358506248	RPC	CellType (Predict)	Neurogenic Cell	Homo sapiens	atonal bHLH transcription factor 7
COL9A2 (ENSG00000049089)	71.435788	-4.2266804	1.0099646	-4.184979	0.00002851925371079	0.0092358506248	RPC	CellType (Predict)	Neurogenic Cell	Homo sapiens	collagen type IX alpha 2 chain
SPSB1 (ENSG00000171621)	51.284454	-3.6950720	0.8899020	-4.152224	0.00003292601520826	0.0103963893020	RPC	CellType (Predict)	Neurogenic Cell	Homo sapiens	splA/ryanodine receptor domain and SOCS box containing 1
- (ENSG00000281938)	153.006412	2.6247962	0.6371897	4.119333	0.00003799712306654	0.0109906678470	RPC	CellType (Predict)	Neurogenic Cell	Mus musculus	novel transcript
LNX1 (ENSG00000072201)	6.424873	6.9003826	1.6770704	4.114546	0.00003879427240087	0.0109922383268	RPC	CellType (Predict)	Neurogenic Cell	Mus musculus	ligand of numb-protein X 1
SV2B (ENSG00000185518)	142.274138	3.5026509	0.8538292	4.102285	0.00004090891844518	0.0113595884739	RPC	CellType (Predict)	Neurogenic Cell	Mus musculus	synaptic vesicle glycoprotein 2B
SCN3A (ENSG00000153253)	78.030668	4.0349221	0.9862964	4.090983	0.00004295483617689	0.0114642704727	RPC	CellType (Predict)	Neurogenic Cell	Mus musculus	sodium voltage-gated channel alpha subunit 3
PCDH9 (ENSG00000184226)	150.002675	-3.7861038	0.9187883	-4.120757	0.00003776289710777	0.0116328144017	RPC	CellType (Predict)	Neurogenic Cell	Homo sapiens	protocadherin 9
ANXA1 (ENSG00000135046)	1,347.359780	-4.9995585	1.2212710	-4.093734	0.00004244811717481	0.0127647552361	RPC	CellType (Predict)	Neurogenic Cell	Homo sapiens	annexin A1
CEBPB (ENSG00000172216)	804.159563	-3.3061336	0.8095733	-4.083798	0.00004430556661957	0.0130134722420	RPC	CellType (Predict)	Neurogenic Cell	Homo sapiens	CCAAT enhancer binding protein beta
ELK3 (ENSG00000111145)	70.723941	-2.9650249	0.7285727	-4.069635	0.00004708692071839	0.0133805954784	RPC	CellType (Predict)	Neurogenic Cell	Homo sapiens	ETS transcription factor ELK3
ANXA2 (ENSG00000182718)	1,079.199646	-3.0637493	0.7533664	-4.066745	0.00004767433068322	0.0133805954784	RPC	CellType (Predict)	Neurogenic Cell	Homo sapiens	annexin A2
FAM78B (ENSG00000188859)	30.783853	3.2623335	0.8091750	4.031678	0.00005537993523816	0.0135415924121	RPC	CellType (Predict)	Neurogenic Cell	Mus musculus	family with sequence similarity 78 member B
KCNH6 (ENSG00000173826)	41.707964	4.7032205	1.1644710	4.038933	0.00005369488594946	0.0135415924121	RPC	CellType (Predict)	Neurogenic Cell	Mus musculus	potassium voltage-gated channel subfamily H member 6
NA (ENSMUSG00000090963)	14.603091	-2.6752125	0.6636975	-4.030771	0.00005559426444044	0.0135415924121	RPC	CellType (Predict)	Neurogenic Cell	Mus musculus	
SOD3 (ENSG00000109610)	420.805491	-5.1442034	1.2714358	-4.045980	0.00005210477559933	0.0137566110216	RPC	CellType (Predict)	Neurogenic Cell	Homo sapiens	superoxide dismutase 3
PPP1R3B (ENSG00000173281)	33.039321	-3.4189608	0.8446358	-4.047852	0.00005168982350646	0.0137566110216	RPC	CellType (Predict)	Neurogenic Cell	Homo sapiens	protein phosphatase 1 regulatory subunit 3B
C1R (ENSG00000159403)	347.963747	-4.1941307	1.0368203	-4.045186	0.00005228165708926	0.0137566110216	RPC	CellType (Predict)	Neurogenic Cell	Homo sapiens	complement C1r
ZFP36 (ENSG00000128016)	2,733.574637	-3.4081142	0.8452887	-4.031894	0.00005532915558783	0.0142613721444	RPC	CellType (Predict)	Neurogenic Cell	Homo sapiens	ZFP36 ring finger protein
PPEF2 (ENSG00000156194)	62.026356	3.7636074	0.9410795	3.999245	0.00006354488992424	0.0149535127408	RPC	CellType (Predict)	Neurogenic Cell	Mus musculus	protein phosphatase with EF-hand domain 2
PKIB (ENSG00000135549)	86.356415	3.3727536	0.8446689	3.992989	0.00006524563599553	0.0149742364651	RPC	CellType (Predict)	Neurogenic Cell	Mus musculus	cAMP-dependent protein kinase inhibitor beta
POMK (ENSG00000185900)	36.790940	-1.6757005	0.4202741	-3.987161	0.00006686852930263	0.0149742364651	RPC	CellType (Predict)	Neurogenic Cell	Mus musculus	protein O-mannose kinase
ST18 (ENSG00000147488)	60.786237	5.2162995	1.3072183	3.990381	0.00006596714630923	0.0149742364651	RPC	CellType (Predict)	Neurogenic Cell	Mus musculus	ST18 C2H2C-type zinc finger transcription factor
CELSR3 (ENSG00000008300)	32.965899	3.2133080	0.8092571	3.970689	0.00007166517133556	0.0157936387115	RPC	CellType (Predict)	Neurogenic Cell	Mus musculus	cadherin EGF LAG seven-pass G-type receptor 3
ADAMTSL1 (ENSG00000178031)	30.755739	-4.3098947	1.0911133	-3.949998	0.00007815199777773	0.0166932667253	RPC	CellType (Predict)	Neurogenic Cell	Mus musculus	ADAMTS like 1
ONECUT2 (ENSG00000119547)	495.036537	3.2343345	0.8199489	3.944556	0.00007994793221750	0.0167609605206	RPC	CellType (Predict)	Neurogenic Cell	Mus musculus	one cut homeobox 2
HDAC9 (ENSG00000048052)	26.951160	3.1703315	0.8042918	3.941768	0.00008088334448878	0.0167609605206	RPC	CellType (Predict)	Neurogenic Cell	Mus musculus	histone deacetylase 9
MT1M (ENSG00000205364)	344.943045	-5.1574291	1.2949613	-3.982690	0.00006813963284409	0.0168745796632	RPC	CellType (Predict)	Neurogenic Cell	Homo sapiens	metallothionein 1M
LMF2 (ENSG00000100258)	58.416833	-1.4217762	0.3614879	-3.933123	0.00008384937527336	0.0169183718122	RPC	CellType (Predict)	Neurogenic Cell	Mus musculus	lipase maturation factor 2
TMEM35A (ENSG00000126950)	101.493983	2.5461594	0.6474720	3.932463	0.00008408006734669	0.0169183718122	RPC	CellType (Predict)	Neurogenic Cell	Mus musculus	transmembrane protein 35A
GPR155 (ENSG00000163328)	26.014611	2.3967303	0.6111300	3.921801	0.00008788956325393	0.0174322670888	RPC	CellType (Predict)	Neurogenic Cell	Mus musculus	G protein-coupled receptor 155
TTBK1 (ENSG00000146216)	7.719667	3.5221296	0.8998985	3.913919	0.00009081014754617	0.0176306878726	RPC	CellType (Predict)	Neurogenic Cell	Mus musculus	tau tubulin kinase 1
GPR135 (ENSG00000181619)	18.009806	-2.8301810	0.7245253	-3.906255	0.00009373745689464	0.0178280938565	RPC	CellType (Predict)	Neurogenic Cell	Mus musculus	G protein-coupled receptor 135
CDKL3 (ENSG00000006837)	35.322778	-1.9267412	0.4953523	-3.889638	0.00010039395795860	0.0179537064107	RPC	CellType (Predict)	Neurogenic Cell	Mus musculus	cyclin dependent kinase like 3
OTX2 (ENSG00000165588)	863.287850	3.4148771	0.8773747	3.892154	0.00009935830744223	0.0179537064107	RPC	CellType (Predict)	Neurogenic Cell	Mus musculus	orthodenticle homeobox 2
TRIM45 (ENSG00000134253)	14.160191	-1.7102323	0.4390027	-3.895722	0.00009790675135041	0.0179537064107	RPC	CellType (Predict)	Neurogenic Cell	Mus musculus	tripartite motif containing 45
ZXDB (ENSG00000198455)	20.951127	-1.9825941	0.5092771	-3.892958	0.00009902940461382	0.0179537064107	RPC	CellType (Predict)	Neurogenic Cell	Mus musculus	zinc finger X-linked duplicated B
KIF3A (ENSG00000131437)	285.222828	-1.0492452	0.2706323	-3.877014	0.00010574641479788	0.0185845977602	RPC	CellType (Predict)	Neurogenic Cell	Mus musculus	kinesin family member 3A
APOE (ENSG00000130203)	2,331.316088	-5.2872253	1.3375767	-3.952839	0.00007722935924004	0.0187578232154	RPC	CellType (Predict)	Neurogenic Cell	Homo sapiens	apolipoprotein E
PPP1R15A (ENSG00000087074)	1,717.974599	-2.1280615	0.5397288	-3.942835	0.00008052421205121	0.0191890716643	RPC	CellType (Predict)	Neurogenic Cell	Homo sapiens	protein phosphatase 1 regulatory subunit 15A
ADAMTSL5 (ENSG00000185761)	29.294406	-3.9111374	1.0129097	-3.861289	0.00011279025413167	0.0195747486046	RPC	CellType (Predict)	Neurogenic Cell	Mus musculus	ADAMTS like 5
EFNA1 (ENSG00000169242)	165.116640	-3.0379542	0.7741099	-3.924448	0.00008692866520954	0.0200605726929	RPC	CellType (Predict)	Neurogenic Cell	Homo sapiens	ephrin A1

Gene	baseMean	log2FoldChange	lfcSE	stat	pvalue	padj	Against	Group	Base	Organism	Gene Name
RHOC (ENSG00000155366)	503.831115	-2.3664980	0.6031965	-3.923262	0.00008735799668334	0.0200605726929	RPC	CellType (Predict)	Neurogenic Cell	Homo sapiens	ras homolog family member C
HEPACAM2 (ENSG00000188175)	11.669113	5.7577323	1.4973596	3.845257	0.00012042626779446	0.0203125918280	RPC	CellType (Predict)	Neurogenic Cell	Mus musculus	HEPACAM family member 2
CHRNB4 (ENSG00000117971)	39.233215	3.6959513	0.9616810	3.843219	0.00012143079240324	0.0203125918280	RPC	CellType (Predict)	Neurogenic Cell	Mus musculus	cholinergic receptor nicotinic beta 4 subunit
MARCHF4 (ENSG00000144583)	14.481572	4.4955617	1.1681827	3.848338	0.00011892202460256	0.0203125918280	RPC	CellType (Predict)	Neurogenic Cell	Mus musculus	membrane associated ring-CH-type finger 4
RAB26 (ENSG00000167964)	16.684910	3.4352254	0.8781897	3.911712	0.00009164404942101	0.0206690061462	RPC	CellType (Predict)	Neurogenic Cell	Homo sapiens	RAB26, member RAS oncogene family
MYBL1 (ENSG00000185697)	57.339297	2.7614465	0.7235448	3.816552	0.00013532934298065	0.0223680071184	RPC	CellType (Predict)	Neurogenic Cell	Mus musculus	MYB proto-oncogene like 1
CHGB (ENSG00000089199)	94.989386	4.1030509	1.0566784	3.882970	0.00010318810132270	0.0228643108720	RPC	CellType (Predict)	Neurogenic Cell	Homo sapiens	chromogranin B
GDAP1 (ENSG00000104381)	117.158255	2.1049124	0.5535557	3.802530	0.00014322568661034	0.0231226213128	RPC	CellType (Predict)	Neurogenic Cell	Mus musculus	ganglioside induced differentiation associated protein 1
LGALS3 (ENSG00000131981)	939.567176	-3.7546143	0.9693210	-3.873448	0.00010730640929841	0.0233668956800	RPC	CellType (Predict)	Neurogenic Cell	Homo sapiens	galectin 3
CAPN2 (ENSG00000162909)	160.054283	-1.5979634	0.4141792	-3.858145	0.00011425071716115	0.0244521009537	RPC	CellType (Predict)	Neurogenic Cell	Homo sapiens	calpain 2
BHLHE41 (ENSG00000123095)	226.942950	-4.3849207	1.1377325	-3.854088	0.00011616199977994	0.0244521009537	RPC	CellType (Predict)	Neurogenic Cell	Homo sapiens	basic helix-loop-helix family member e41
CA12 (ENSG00000074410)	55.519063	-4.7217719	1.2286802	-3.842963	0.00012155804536903	0.0251684936559	RPC	CellType (Predict)	Neurogenic Cell	Homo sapiens	carbonic anhydrase 12
SH3BP2 (ENSG00000087266)	54.641722	-2.7759075	0.7246078	-3.830910	0.00012767006176953	0.0255948076214	RPC	CellType (Predict)	Neurogenic Cell	Homo sapiens	SH3 domain binding protein 2
SOCS3 (ENSG00000184557)	823.473930	-3.6402926	0.9495421	-3.833735	0.00012621229594833	0.0255948076214	RPC	CellType (Predict)	Neurogenic Cell	Homo sapiens	suppressor of cytokine signaling 3
LHX4 (ENSG00000121454)	149.652484	4.2884330	1.1383150	3.767352	0.00016498846036789	0.0257685148269	RPC	CellType (Predict)	Neurogenic Cell	Mus musculus	LIM homeobox 4
PLD2 (ENSG00000129219)	22.120611	-2.6545994	0.7046877	-3.767058	0.00016518278735186	0.0257685148269	RPC	CellType (Predict)	Neurogenic Cell	Mus musculus	phospholipase D2
FBXW4 (ENSG00000107829)	10.174283	-2.3538810	0.6263778	-3.757926	0.00017132782371129	0.0261397308177	RPC	CellType (Predict)	Neurogenic Cell	Mus musculus	F-box and WD repeat domain containing 4
LYVE1 (ENSG00000133800)	76.766417	5.1445941	1.3688486	3.758337	0.00017104651803253	0.0261397308177	RPC	CellType (Predict)	Neurogenic Cell	Mus musculus	lymphatic vessel endothelial hyaluronan receptor 1
EOMES (ENSG00000163508)	15.147312	5.3396233	1.4243468	3.748823	0.00017766671037535	0.0263054407400	RPC	CellType (Predict)	Neurogenic Cell	Mus musculus	eomesodermin
SCAP (ENSG00000114650)	101.043380	-1.0944047	0.2919803	-3.748215	0.00017809791339365	0.0263054407400	RPC	CellType (Predict)	Neurogenic Cell	Mus musculus	SREBF chaperone
HNRNPUL2 (ENSG00000214753)	85.293614	-1.0270408	0.2738827	-3.749930	0.00017688400546503	0.0263054407400	RPC	CellType (Predict)	Neurogenic Cell	Mus musculus	heterogeneous nuclear ribonucleoprotein U like 2
ZNF583 (ENSG00000198440)	13.662769	-2.1679557	0.5788487	-3.745289	0.00018018606129278	0.0263337186841	RPC	CellType (Predict)	Neurogenic Cell	Mus musculus	zinc finger protein 583
YIPF1 (ENSG00000058799)	162.335752	-0.6877391	0.1838068	-3.741641	0.00018282243722519	0.0264406949837	RPC	CellType (Predict)	Neurogenic Cell	Mus musculus	Yip1 domain family member 1
MT2A (ENSG00000125148)	3,398.990718	-3.1948049	0.8368274	-3.817759	0.00013466955397047	0.0265761947914	RPC	CellType (Predict)	Neurogenic Cell	Homo sapiens	metallothionein 2A
ATOH7 (ENSG00000179774)	839.162658	3.7229446	0.9972052	3.733379	0.00018892820212316	0.0270420531781	RPC	CellType (Predict)	Neurogenic Cell	Mus musculus	atonal bHLH transcription factor 7
ZNF641 (ENSG00000167528)	14.027331	-2.5715751	0.6905843	-3.723767	0.00019627184643987	0.0278065134283	RPC	CellType (Predict)	Neurogenic Cell	Mus musculus	zinc finger protein 641
GMIP (ENSG00000089639)	15.053778	2.3791833	0.6284545	3.785768	0.00015323436793246	0.0281298592132	RPC	CellType (Predict)	Neurogenic Cell	Homo sapiens	GEM interacting protein
SRRM4 (ENSG00000139767)	104.837256	3.4820825	0.9176671	3.794494	0.00014794473709695	0.0281298592132	RPC	CellType (Predict)	Neurogenic Cell	Homo sapiens	serine/arginine repetitive matrix 4
GYPC (ENSG00000136732)	420.669155	-3.4235419	0.9037889	-3.787988	0.00015187188989816	0.0281298592132	RPC	CellType (Predict)	Neurogenic Cell	Homo sapiens	glycophorin C (Gerbich blood group)
CCL2 (ENSG00000108691)	752.460061	-5.1991496	1.3736018	-3.785049	0.00015367856577309	0.0281298592132	RPC	CellType (Predict)	Neurogenic Cell	Homo sapiens	C-C motif chemokine ligand 2
ZFAND1 (ENSG00000104231)	114.305909	-0.8857533	0.2387060	-3.710646	0.00020673126301056	0.0284183847093	RPC	CellType (Predict)	Neurogenic Cell	Mus musculus	zinc finger AN1-type containing 1
LIMA1 (ENSG00000050405)	15.869651	-3.0573055	0.8238437	-3.711026	0.00020642065461962	0.0284183847093	RPC	CellType (Predict)	Neurogenic Cell	Mus musculus	LIM domain and actin binding 1
COL9A3 (ENSG00000092758)	94.172878	-3.3340934	0.8825248	-3.777903	0.00015815415547589	0.0285355283380	RPC	CellType (Predict)	Neurogenic Cell	Homo sapiens	collagen type IX alpha 3 chain
DNAJC3 (ENSG00000102580)	289.486335	-1.2369582	0.3282771	-3.768031	0.00016454043418002	0.0292696575168	RPC	CellType (Predict)	Neurogenic Cell	Homo sapiens	DnaJ heat shock protein family (Hsp40) member C3
JRK (ENSG00000234616)	18.292895	-1.4468878	0.3929648	-3.681978	0.00023143131957179	0.0315018866758	RPC	CellType (Predict)	Neurogenic Cell	Mus musculus	Jrk helix-turn-helix protein
SNAP25 (ENSG00000132639)	2,046.603757	3.1259941	0.8504986	3.675484	0.00023739866170582	0.0317384360802	RPC	CellType (Predict)	Neurogenic Cell	Mus musculus	synaptosome associated protein 25
BBS10 (ENSG00000179941)	18.842056	-1.8061364	0.4914501	-3.675116	0.00023774109423338	0.0317384360802	RPC	CellType (Predict)	Neurogenic Cell	Mus musculus	Bardet-Biedl syndrome 10
SEZ6L2 (ENSG00000174938)	48.289735	3.2788696	0.8933446	3.670330	0.00024223740920315	0.0318396127501	RPC	CellType (Predict)	Neurogenic Cell	Mus musculus	seizure related 6 homolog like 2
PHLDA1 (ENSG00000139289)	774.977376	2.6803470	0.7304519	3.669437	0.00024308549060139	0.0318396127501	RPC	CellType (Predict)	Neurogenic Cell	Mus musculus	pleckstrin homology like domain family A member 1
NA (ENSMUSG00000071302)	34.267731	-0.9085127	0.2479251	-3.664464	0.00024785731919150	0.0318748157724	RPC	CellType (Predict)	Neurogenic Cell	Mus musculus	
TMEM63A (ENSG00000196187)	44.362184	-3.5192078	0.9631576	-3.653823	0.00025836400205088	0.0329094110502	RPC	CellType (Predict)	Neurogenic Cell	Mus musculus	transmembrane protein 63A
FBLIM1 (ENSG00000162458)	47.457591	2.8176183	0.7558628	3.727685	0.00019324704450965	0.0334343859200	RPC	CellType (Predict)	Neurogenic Cell	Homo sapiens	filamin binding LIM protein 1
GLUL (ENSG00000135821)	2,391.084014	-2.7168912	0.7282911	-3.730502	0.00019109899591720	0.0334343859200	RPC	CellType (Predict)	Neurogenic Cell	Homo sapiens	glutamate-ammonia ligase
CPLX2 (ENSG00000145920)	30.121980	4.8574122	1.3072480	3.715754	0.00020259842868528	0.0345786237067	RPC	CellType (Predict)	Neurogenic Cell	Homo sapiens	complexin 2
NAGK (ENSG00000124357)	135.717864	-0.9735057	0.2679027	-3.633803	0.00027927407445220	0.0349319031504	RPC	CellType (Predict)	Neurogenic Cell	Mus musculus	N-acetylglucosamine kinase
COCH (ENSG00000100473)	35.471650	-3.2367904	0.8913702	-3.631253	0.00028204895627722	0.0349639974014	RPC	CellType (Predict)	Neurogenic Cell	Mus musculus	cochlin

Gene	baseMean	log2FoldChange	lfcSE	stat	pvalue	padj	Against	Group	Base	Organism	Gene Name
MYBL1 (ENSG00000185697)	17.294167	2.2926101	0.6191916	3.702586	0.00021341308713524	0.0359387638736	RPC	CellType (Predict)	Neurogenic Cell	Homo sapiens	MYB proto-oncogene like 1
ACTL6B (ENSG00000077080)	99.737466	3.0906425	0.8549259	3.615100	0.00030023194645300	0.0362471334309	RPC	CellType (Predict)	Neurogenic Cell	Mus musculus	actin like 6B
PPP1R14A (ENSG00000167641)	178.507565	3.2168963	0.8893654	3.617069	0.00029795744219601	0.0362471334309	RPC	CellType (Predict)	Neurogenic Cell	Mus musculus	protein phosphatase 1 regulatory inhibitor subunit 14A
TRIP6 (ENSG00000087077)	121.207385	-1.9173204	0.5187799	-3.695827	0.00021917247444955	0.0364230046355	RPC	CellType (Predict)	Neurogenic Cell	Homo sapiens	thyroid hormone receptor interactor 6
SAT1 (ENSG00000130066)	3,782.497089	-2.1956462	0.5947243	-3.691872	0.00022260937192001	0.0365137190565	RPC	CellType (Predict)	Neurogenic Cell	Homo sapiens	spermidine/spermine N1-acetyltransferase 1
NA (ENSG00000148671)	1,029.384794	-4.5908617	1.2454763	-3.686029	0.00022778051447417	0.0368829217668	RPC	CellType (Predict)	Neurogenic Cell	Homo sapiens	
RNPEPL1 (ENSG00000142327)	91.243803	-0.9428048	0.2618931	-3.599960	0.00031826567831379	0.0380931092906	RPC	CellType (Predict)	Neurogenic Cell	Mus musculus	arginyl aminopeptidase like 1
HS3ST1 (ENSG00000002587)	64.096938	2.6824408	0.7464154	3.593764	0.00032593528813751	0.0386776541923	RPC	CellType (Predict)	Neurogenic Cell	Mus musculus	heparan sulfate-glucosamine 3-sulfotransferase 1
PER1 (ENSG00000179094)	106.646978	-2.0588353	0.5609041	-3.670566	0.00024201440264409	0.0386916696885	RPC	CellType (Predict)	Neurogenic Cell	Homo sapiens	period circadian regulator 1
RFC2 (ENSG00000049541)	215.063699	-0.6788928	0.1890753	-3.590596	0.00032992296554987	0.0387861600051	RPC	CellType (Predict)	Neurogenic Cell	Mus musculus	replication factor C subunit 2
BIVM (ENSG00000134897)	39.561127	-1.0703473	0.2982618	-3.588617	0.00033243683669038	0.0387861600051	RPC	CellType (Predict)	Neurogenic Cell	Mus musculus	basic, immunoglobulin-like variable motif containing
DCC (ENSG00000187323)	120.925589	3.3903083	0.9475984	3.577790	0.00034651120691824	0.0397600131971	RPC	CellType (Predict)	Neurogenic Cell	Mus musculus	DCC netrin 1 receptor
MAP2K3 (ENSG00000034152)	154.537096	-2.1018436	0.5743753	-3.659356	0.00025284977163793	0.0399186576973	RPC	CellType (Predict)	Neurogenic Cell	Homo sapiens	mitogen-activated protein kinase kinase 3
PROZ (ENSG00000126231)	9.135586	2.8016246	0.7907756	3.542882	0.00039577962095397	0.0406019340611	RPC	CellType (Predict)	Neurogenic Cell	Mus musculus	protein Z, vitamin K dependent plasma glycoprotein
CARMIL2 (ENSG00000159753)	17.424027	3.5094179	0.9885156	3.550190	0.00038495345597958	0.0406019340611	RPC	CellType (Predict)	Neurogenic Cell	Mus musculus	capping protein regulator and myosin 1 linker 2
INPP1 (ENSG00000151689)	103.565536	-1.4125653	0.3981189	-3.548099	0.00038802232132340	0.0406019340611	RPC	CellType (Predict)	Neurogenic Cell	Mus musculus	inositol polyphosphate-1-phosphatase
ABCD2 (ENSG00000173208)	93.300488	2.0191519	0.5686299	3.550907	0.00038390602669197	0.0406019340611	RPC	CellType (Predict)	Neurogenic Cell	Mus musculus	ATP binding cassette subfamily D member 2
ELAVL3 (ENSG00000196361)	248.724180	3.4359102	0.9669907	3.553199	0.00038057687423562	0.0406019340611	RPC	CellType (Predict)	Neurogenic Cell	Mus musculus	ELAV like RNA binding protein 3
COL11A2 (ENSG00000204248)	32.637860	-2.9028764	0.8161957	-3.556594	0.00037569478659711	0.0406019340611	RPC	CellType (Predict)	Neurogenic Cell	Mus musculus	collagen type XI alpha 2 chain
ATXN1L (ENSG00000224470)	35.601819	-1.1410700	0.3221909	-3.541596	0.00039771413370149	0.0406019340611	RPC	CellType (Predict)	Neurogenic Cell	Mus musculus	ataxin 1 like
NEUROD4 (ENSG00000123307)	509.923549	4.5102932	1.2723061	3.544975	0.00039265054906460	0.0406019340611	RPC	CellType (Predict)	Neurogenic Cell	Mus musculus	neuronal differentiation 4
GET1 (ENSG00000182093)	211.501437	-0.7637907	0.2155263	-3.543840	0.00039434405196984	0.0406019340611	RPC	CellType (Predict)	Neurogenic Cell	Mus musculus	guided entry of tail-anchored proteins factor 1
STOM (ENSG00000148175)	215.412995	-2.9848952	0.8183517	-3.647448	0.00026485781846468	0.0411197908558	RPC	CellType (Predict)	Neurogenic Cell	Homo sapiens	stomatin
PSD4 (ENSG00000125637)	27.520452	-3.3571448	0.9209247	-3.645407	0.00026696934680701	0.0411197908558	RPC	CellType (Predict)	Neurogenic Cell	Homo sapiens	pleckstrin and Sec7 domain containing 4
ILVBL (ENSG00000105135)	112.137075	-0.8525961	0.2412917	-3.533466	0.00041014912905921	0.0412645688975	RPC	CellType (Predict)	Neurogenic Cell	Mus musculus	ilvB acetolactate synthase like
CARD6 (ENSG00000132357)	10.071514	-3.1612188	0.8960795	-3.527833	0.00041897623746470	0.0415504720069	RPC	CellType (Predict)	Neurogenic Cell	Mus musculus	caspase recruitment domain family member 6
LMO4 (ENSG00000143013)	372.622075	-1.9872287	0.5465186	-3.636159	0.00027673334513478	0.0421101463741	RPC	CellType (Predict)	Neurogenic Cell	Homo sapiens	LIM domain only 4
CHRNA3 (ENSG00000080644)	106.614471	3.1148261	0.8876977	3.508881	0.00044995549643786	0.0433373058857	RPC	CellType (Predict)	Neurogenic Cell	Mus musculus	cholinergic receptor nicotinic alpha 3 subunit
IFNGR1 (ENSG00000027697)	339.055504	-1.0913266	0.3108679	-3.510580	0.00044713011213040	0.0433373058857	RPC	CellType (Predict)	Neurogenic Cell	Mus musculus	interferon gamma receptor 1
NYAP2 (ENSG00000144460)	60.409074	3.9179208	1.1154575	3.512389	0.00044409723245668	0.0433373058857	RPC	CellType (Predict)	Neurogenic Cell	Mus musculus	neuronal tyrosine-phosphorylated phosphoinositide-3-kinase adaptor 2
MAFB (ENSG00000204103)	496.703031	-3.0821436	0.8517637	-3.618543	0.00029626645794772	0.0437802564090	RPC	CellType (Predict)	Neurogenic Cell	Homo sapiens	MAF bZIP transcription factor B
SLCO1C1 (ENSG00000139155)	15.318924	-5.6359893	1.5582208	-3.616939	0.00029810784253167	0.0437802564090	RPC	CellType (Predict)	Neurogenic Cell	Homo sapiens	solute carrier organic anion transporter family member 1C1
SUMF2 (ENSG00000129103)	71.135580	-1.0845751	0.3102651	-3.495640	0.00047292664828837	0.0443656323300	RPC	CellType (Predict)	Neurogenic Cell	Mus musculus	sulfatase modifying factor 2
ARL6IP5 (ENSG00000144746)	550.303598	-0.7294707	0.2086207	-3.496637	0.00047116268338640	0.0443656323300	RPC	CellType (Predict)	Neurogenic Cell	Mus musculus	ADP ribosylation factor like GTPase 6 interacting protein 5
QSOX1 (ENSG00000116260)	167.737100	2.1218892	0.6065441	3.498326	0.00046818768231648	0.0443656323300	RPC	CellType (Predict)	Neurogenic Cell	Mus musculus	quiescin sulfhydryl oxidase 1
PPP1R3G (ENSG00000219607)	8.130249	-3.4218412	0.9489414	-3.605956	0.00031100548874574	0.0451494175041	RPC	CellType (Predict)	Neurogenic Cell	Homo sapiens	protein phosphatase 1 regulatory subunit 3G
STAT6 (ENSG00000166888)	59.932524	2.0819011	0.5782254	3.600501	0.00031760460391627	0.0455834789484	RPC	CellType (Predict)	Neurogenic Cell	Homo sapiens	signal transducer and activator of transcription 6
SEZ6L2 (ENSG00000174938)	156.246116	3.6558234	1.0162084	3.597513	0.00032127385352681	0.0455920086522	RPC	CellType (Predict)	Neurogenic Cell	Homo sapiens	seizure related 6 homolog like 2
PMEPA1 (ENSG00000124225)	364.979005	-2.0802726	0.5967557	-3.485970	0.00049035519902603	0.0456918898207	RPC	CellType (Predict)	Neurogenic Cell	Mus musculus	prostate transmembrane protein, androgen induced 1
FGFBP3 (ENSG00000174721)	42.972710	-1.8189079	0.5061537	-3.593588	0.00032615502723011	0.0457704221546	RPC	CellType (Predict)	Neurogenic Cell	Homo sapiens	fibroblast growth factor binding protein 3
MLC1 (ENSG00000100427)	14.225327	-5.2161160	1.4582554	-3.576956	0.00034761822489901	0.0482463536316	RPC	CellType (Predict)	Neurogenic Cell	Homo sapiens	modulator of VRAC current 1
RARA (ENSG00000131759)	97.828308	-1.3039503	0.3763754	-3.464494	0.00053122966856478	0.0485236363050	RPC	CellType (Predict)	Neurogenic Cell	Mus musculus	retinoic acid receptor alpha
SCRT2 (ENSG00000215397)	39.334643	3.4903785	1.0082684	3.461755	0.00053666514277380	0.0486294325808	RPC	CellType (Predict)	Neurogenic Cell	Mus musculus	scratch family transcriptional repressor 2
PWWP2B (ENSG00000171813)	41.324210	2.0722097	0.5994391	3.456915	0.00054639811888510	0.0486294325808	RPC	CellType (Predict)	Neurogenic Cell	Mus musculus	PWWP domain containing 2B
ABCG4 (ENSG00000172350)	38.094129	-2.1533415	0.6224765	-3.459314	0.00054155386699652	0.0486294325808	RPC	CellType (Predict)	Neurogenic Cell	Mus musculus	ATP binding cassette subfamily G member 4
TRIM62 (ENSG00000116525)	23.069509	2.4587519	0.7111733	3.457317	0.00054558198324721	0.0486294325808	RPC	CellType (Predict)	Neurogenic Cell	Mus musculus	tripartite motif containing 62

Gene	baseMean	log2FoldChange	lfcSE	stat	pvalue	padj	Against	Group	Base	Organism	Gene Name
PMP22 (ENSG00000109099)	447.125671	-3.3870277	0.9491726	-3.568400	0.00035916756251928	0.0487772722002	RPC	CellType (Predict)	Neurogenic Cell	Homo sapiens	peripheral myelin protein 22
NA (ENSG00000133321)	173.282438	-4.1667608	1.1674365	-3.569154	0.00035813558668590	0.0487772722002	RPC	CellType (Predict)	Neurogenic Cell	Homo sapiens	
ARID5B (ENSG00000150347)	522.120429	-2.6624348	0.7485977	-3.556563	0.00037573858543111	0.0489885413743	RPC	CellType (Predict)	Neurogenic Cell	Homo sapiens	AT-rich interaction domain 5B
ASAH1 (ENSG00000104763)	405.710670	-1.4604737	0.4105371	-3.557471	0.00037444269876531	0.0489885413743	RPC	CellType (Predict)	Neurogenic Cell	Homo sapiens	N-acylsphingosine amidohydrolase 1
PRELP (ENSG00000188783)	239.486722	-4.6333944	1.3048707	-3.550846	0.00038399569248283	0.0489885413743	RPC	CellType (Predict)	Neurogenic Cell	Homo sapiens	proline and arginine rich end leucine rich repeat protein
SYTL2 (ENSG00000137501)	41.885793	-3.1140905	0.8768627	-3.551400	0.00038318689469553	0.0489885413743	RPC	CellType (Predict)	Neurogenic Cell	Homo sapiens	synaptotagmin like 2
CYRIA (ENSG00000197872)	11.627584	3.9168659	1.1010078	3.557528	0.00037436140897300	0.0489885413743	RPC	CellType (Predict)	Neurogenic Cell	Homo sapiens	CYFIP related Rac1 interactor A
PDPN (ENSG00000162493)	120.834219	-4.4270558	1.2450339	-3.555771	0.00037687187235611	0.0489885413743	RPC	CellType (Predict)	Neurogenic Cell	Homo sapiens	podoplanin

Supplemental Table 5: Differentially expressed genes between RPC and neurogenic cells in human and mouse. Genes filtered with a abs(log2FoldChange) > 2 and a padj < 0.01.

Gene	Ensembl	baseMean	log2FoldChange	lfcSE	stat	pvalue	padj
PDE6G	ENSG00000185527	66.333277	-2.3441678	0.4168273	-5.623835	0.00000001867637	0.0000490068
AKAP12	ENSG00000131016	16.211349	3.2204333	0.6509519	4.947268	0.00000075262380	0.0005739686
HSPB1	ENSG00000106211	33.905799	2.7358265	0.5443945	5.025449	0.00000050225706	0.0005739686
GADD45B	ENSG00000099860	10.407572	2.7951199	0.5683610	4.917861	0.00000087495220	0.0005739686
CC2D2A	ENSG00000048342	65.778406	-1.8123582	0.4122057	-4.396733	0.00001098925099	0.0057671589
CALB1	ENSG00000104327	18.454365	2.8703664	0.6662940	4.307958	0.00001647686745	0.0072058834
RP1L1	ENSG00000183638	27.760158	-2.0706619	0.4881859	-4.241544	0.00002219871789	0.0083213480
RRAD	ENSG00000166592	10.848913	2.8671937	0.6933500	4.135276	0.00003545284876	0.0116285344
H2AJ	ENSG00000246705	14.256520	-1.7133388	0.4374781	-3.916399	0.00008988147974	0.0262054448
GNGT2	ENSG00000167083	21.586045	-1.8518370	0.4832323	-3.832188	0.00012700851355	0.0333270340
SLC24A4	ENSG00000140090	12.091555	-2.3479519	0.6275797	-3.741281	0.00018308493710	0.0391603991
EIF3E	ENSG00000104408	25.640591	1.7284801	0.4593680	3.762735	0.00016806515137	0.0391603991
GPM6A	ENSG00000150625	14.342849	2.0169193	0.5412094	3.726690	0.00019401112370	0.0391603991
SLC6A8	ENSG00000130821	18.194596	-1.5484910	0.4190325	-3.695395	0.00021954477814	0.0411489641
ATP8	ENSG00000228253	24.808372	-2.3805564	0.6505116	-3.659514	0.00025269395502	0.0414418086
JUN	ENSG00000177606	103.963097	2.0746674	0.5646982	3.673940	0.00023883868696	0.0414418086
LBH	ENSG00000213626	22.178216	-1.5820553	0.4438071	-3.564736	0.00036422213745	0.0562187582
MARCKS	ENSG00000277443	38.040849	1.4382002	0.4104345	3.504092	0.00045816776633	0.0632753799
ATP8A2	ENSG00000132932	15.822891	-1.7063362	0.4867345	-3.505681	0.00045543997512	0.0632753799
SORL1	ENSG00000137642	9.295426	-2.0038012	0.5777338	-3.468382	0.00052360288684	0.0654254274
IGF1R	ENSG00000140443	10.280407	1.6259991	0.4675512	3.477692	0.00050575055876	0.0654254274
PSIP1	ENSG00000164985	10.390270	1.6926238	0.4991538	3.390987	0.00069641472693	0.0730080016
AP1S2	ENSG00000182287	15.468753	1.3309232	0.3949131	3.370167	0.00075122562584	0.0730080016

Gene	Ensembl	baseMean	log2FoldChange	lfcSE	stat	pvalue	padj
PDLIM1	ENSG00000107438	18.097895	-1.6983405	0.5009208	-3.390437	0.00069781251494	0.0730080016
MCF2	ENSG00000101977	7.817816	-2.5509416	0.7532575	-3.386546	0.00070778317976	0.0730080016
ANKRD33	ENSG00000167612	31.069622	-1.6982079	0.4976874	-3.412198	0.00064441375827	0.0730080016
TRAC	ENSG00000277734	37.916861	-1.4915835	0.4413536	-3.379566	0.00072600465462	0.0730080016
RIMS2	ENSG00000176406	178.998521	-0.9930176	0.2976702	-3.335966	0.00085003661447	0.0796605742
DNAJB1	ENSG00000132002	44.874704	1.1598484	0.3504145	3.309933	0.00093318389388	0.0844370530
FBL	ENSG00000105202	7.563700	1.9835304	0.6023149	3.293178	0.00099061590154	0.0866458709
FOS	ENSG00000170345	65.413599	2.2598093	0.7217566	3.130985	0.00174220778280	0.0982445521
UCKL1	ENSG00000198276	21.260475	-1.4652520	0.4673063	-3.135528	0.00171544977959	0.0982445521
DHRS11	ENSG00000278535	27.726544	-1.4295704	0.4514196	-3.166832	0.00154109089340	0.0982445521
PFKP	ENSG00000067057	9.676249	-2.0719797	0.6594725	-3.141874	0.00167870055349	0.0982445521
NR1D2	ENSG00000174738	15.146679	1.5612863	0.4908196	3.180978	0.00146778954795	0.0982445521
ELOVL6	ENSG00000170522	7.849669	-1.8862844	0.5887784	-3.203726	0.00135661747463	0.0982445521
RAC1	ENSG00000136238	17.670474	1.3239688	0.4090086	3.237019	0.00120785235883	0.0982445521
BAMBI	ENSG00000095739	7.614820	2.6095010	0.8164728	3.196066	0.00139315244017	0.0982445521
C12orf57	ENSG00000111678	29.288427	1.0680875	0.3410052	3.132174	0.00173517129937	0.0982445521
SGK1	ENSG00000118515	15.018077	-1.6407528	0.5122446	-3.203065	0.00135973340152	0.0982445521
KBTBD11	ENSG00000176595	9.955960	-1.8440108	0.5833912	-3.160848	0.00157310538747	0.0982445521
NSA2	ENSG00000164346	13.607928	1.2706136	0.3961188	3.207658	0.00133820604225	0.0982445521
RPL6	ENSG00000089009	42.284446	1.1969586	0.3826535	3.128048	0.00175971568145	0.0982445521
LIMA1	ENSG00000050405	29.288181	-1.4856845	0.4719500	-3.147970	0.00164408359061	0.0982445521
MCL1	ENSG00000143384	11.135773	1.3862735	0.4282330	3.237195	0.00120711049749	0.0982445521
TMEM14B	ENSG00000137210	20.558167	-1.3397530	0.4279596	-3.130559	0.00174473827329	0.0982445521
USP32	ENSG00000170832	17.570164	-1.4198796	0.4481899	-3.168031	0.00153474949030	0.0982445521

Supplemental Table 6: Macula versus peripheral cone differential gene testing table
